# Building multiscale Markov state models by systematic mapping of temporal communities

**DOI:** 10.1093/bioinformatics/btaf585

**Published:** 2025-11-25

**Authors:** Nir Nitskansky, Kessem Clein, Barak Raveh

**Affiliations:** School of Computer Science and Engineering, The Hebrew University of Jerusalem, The Edmond J. Safra Campus, Jerusalem 9190401, Israel; School of Computer Science and Engineering, The Hebrew University of Jerusalem, The Edmond J. Safra Campus, Jerusalem 9190401, Israel; School of Computer Science and Engineering, The Hebrew University of Jerusalem, The Edmond J. Safra Campus, Jerusalem 9190401, Israel

## Abstract

**Motivation:**

Biomolecules undergo dynamic transitions among metastable states to carry out their biological functions. Markov State Models (MSMs) effectively capture these metastable states and transitions at a defined temporal scale. However, biomolecular dynamics typically span multiple temporal scales, ranging from fast atomic vibrations to slower conformational changes and folding events.

**Results:**

We introduce multiscale Markov State Models (mMSMs), which capture biomolecular dynamics across multiple temporal resolutions simultaneously *via* a hierarchy of MSMs, and mMSM-explore, an unsupervised algorithm for generating mMSMs through multiscale adaptive sampling with on-the-fly identification of temporally metastable states. We benchmark our method on a toy system with nested energy minima; on alanine dipeptide, first with and then without assuming prior knowledge of its two reaction coordinates; and finally, on a fast-folding 35-residue miniprotein, where we map folding pathways across scales. We demonstrate efficient mapping of energy landscapes, correct representation of multiscale hierarchies and transition states, accurate inference of stationary probabilities and transition kinetics, as well as *de novo* identification of underlying slow, intermediate, and fast reaction coordinates. mMSMs reveal how dynamic processes at different scales contribute collectively to the functional mechanisms of biomolecular machines.

**Availability and implementation:**

Python code and instructions are available at https://github.com/ravehlab/mMSM.

## 1 Introduction

Molecular dynamics (MD) simulations are used extensively for studying biomolecular processes (Karplus and Petsko 1990, Adcock and McCammon 2006), including protein folding and misfolding (Lemkul and Bevan 2010), characterization of metabolic reactions in cells (Böckmann and Grubmüller 2002), and accelerated drug discovery (Das *et al.* 2021, Szymczak *et al.* 2023). These processes span timescales from picoseconds to minutes (Tozzini 2010, Lindorff-Larsen *et al.* 2011). However, all-atom simulations of large systems are computationally expensive, limiting our ability to simulate large systems over sufficiently long timescales, and to collect sufficient statistics on key processes (Durrant and McCammon 2011, Feig *et al.* 2018, Shaw *et al.* 2021). Advances in MD-optimized hardware (Bailey *et al.* 2017), complemented by computational and theoretical methods (Vanden-Eijnden 2010, Lee *et al.* 2018, Zhu *et al.* 2018, Hénin *et al.* 2022), continue to push these boundaries, with dedicated supercomputers now generating billions to trillions of simulation frames per day, for systems comprising up to hundreds of millions of atoms (Shaw *et al.* 2021).

These advances highlight a key challenge: summarizing the high-dimensional MD data into a compact dynamical representation of the system’s dynamics. Transition state theory (Eyring 1935, Evans and Polanyi 1935) establishes that transition rates decay exponentially with the magnitude of the intervening free energy barrier, trapping biomolecular simulations in local minima with infrequent transitions. A useful representation should thus capture a system’s metastable states, transition rates, and their equilibrium or steady-state probabilities (Kalikow and McCutcheon 2010).

Markov State Models (Grubmüller and Tavan 1994, Husic and Pande 2018) (MSMs) are a popular approach for generating such a representation from either long MD trajectories or multiple short ones—namely, a network of metastable states and transition rates between these states. The MSM methodology commonly leverages adaptive sampling techniques to systematically and efficiently discover new system states and to provide robust statistics for transitions between existing ones, by using the current set of states of the MSM as milestones for subsequent short simulations (Faradjian and Elber 2004, Bowman *et al.* 2010, Schütte *et al.* 2011, Bhoutekar *et al.* 2017, Wan and Voelz 2020, Chan and Shukla 2021). MSMs have been used to characterize the dynamics of diverse systems, including protein folding trajectories (Chodera and Noé 2014), protein-protein interactions (Plattner *et al.* 2017), enzymatic activity (Hart *et al.* 2016, Shukla *et al.* 2014), and folding-upon-binding of intrinsically disordered proteins (Sisk and Robustelli 2024).

A major limitation of MSMs is their non-hierarchical nature. Broadly speaking, MSMs are often constructed by partitioning sampled configurations into a finite set of microstates; estimating transition probabilities at a single, relatively small time resolution, or lag time; and finally, coarse-graining these microstates into kinetically meaningful macrostates associated with processes occurring over longer timescales (Deuflhard and Weber 2005, Röblitz and Weber 2013, Yao *et al.* 2013). However, biomolecular dynamics typically span a wide range of temporal scales, associated with free-energy barriers differing by orders of magnitude (Grimaldo *et al.* 2019, Suárez et al. 2021). For example, protein folding over millisecond timescales involves hydrogen bond vibrations on the picosecond scale, side chain fluctuations over nanoseconds, and conformational transitions over microseconds (Xu and Havenith 2015). Deep learning-based approaches such as VAMPnets coarse-grain MD trajectories to a fixed number of states by maximizing a variational score to preserve the system’s slow dynamical modes at a chosen input granularity, but the resulting states are still associated with a specific time resolution (Mardt *et al.* 2018, Sidky *et al.* 2019). Recent hierarchical schemes partly address this gap by coupling an MSM constructed at one lag time to a coarser-scale kinetic description, such as another MSM linking separated basins (Wolfe *et al.* 2020) or a continuum reaction-diffusion model (Del Razo *et al.* 2021). However, these methods are typically limited to exactly two layers at predefined temporal scales (Table S3, available as supplementary data at *Bioinformatics* online). We propose that a more complete representation of the system’s dynamics should capture the full hierarchy of distinct processes and their respective temporal resolutions.

Here, we introduce the multiscale Markov State Model (mMSM), a new data structure that describes a dynamic biomolecular system as a nested hierarchy of standard MSMs, each defined at its own lag time. To avoid the need to pre-specify lag times, we constrain the ratio between the lag times of adjacent layers, thus enabling unsupervised discovery of underlying processes spanning multiple temporal resolutions. We also present mMSM-explore, an algorithm that iteratively expands an mMSM from an initial configuration, adaptively steering new simulations toward under-explored regions of the configuration space across scales, while simultaneously identifying a hierarchy of temporal communities. By exploiting the mMSM’s multiscale organization, mMSM-explore samples the configuration space efficiently, decoupling the exploration of rapid transitions confined to shallow free-energy basins (e.g. vibrations of water molecules) from progressively slower transitions among deeper basins (e.g. backbone conformational changes).

Algorithm 1 mMSM-explore

 **Input:**  $x_{\mathrm{init}}$, an initial configuration

 **Output:**mmsm, an mMSM data structure

 **Parameters:** 

  $t_{\max}$, maximal accumulated simulation time

   $n_{\mathrm{init}}$, initial configurations per sampling iteration

  *k*, trajectories per initial configuration

  *m*, time steps per trajectory

1: mmsm  ←  NewMultiscaleMSM($x_{\mathrm{init}}$)

2: *X* ← {$x_{\mathrm{init}}$}

3: $t_{\mathrm{sim}}\leftarrow 0$  

4: **while**  $t_{\mathrm{sim}}$ < $t_{\max}$  **do** 

5:   Γ  ←  ∅

6:   **for**  x∈X  **do** 

7:    ${\left\{\gamma_{i}\right\}}_{i=1}^{k}\leftarrow$  Simulator(*x*, *k*, *m*)

8:    $t_{\mathrm{sim}}\leftarrow t_{\mathrm{sim}}+\tau_{0}\cdot k\cdot (m-1)$

9:    $\Gamma =\Gamma \cup {\left\{\gamma_{i}\right\}}_{i=1}^{k}$

10:    X←X∖{x}

11:   **end for** 

12:   UpdateMultiscaleMSM(mmsm, Γ)

13:   **for**  $i\in [1..n_{\mathrm{init}}]$  **do** 

14:    x←  MultiscaleAdaptiveSampling(mmsm)

15:    X←X∪{x}

16:   **end for** 

17: **end while** 

18: **return**mmsm

Algorithm 2 MultiscaleAdaptiveSampling

 **Input:**mmsm, an mMSM data structure

 **Output:** *x*, a system configuration

1: $S_{\text{cur}}$  ←mmsm.root

2: **while**  $S_{\text{cur}}\notin \mathrm{mmsm}.M_{0}$  **do** 

3:  $S_{\text{cur}}\leftarrow$ sample from $S_{\text{cur}}.\mathrm{children}$ using $P_{\text{adap}}$

4: **end while** 

5: $x\leftarrow \text{Representative}(S_{\text{cur}})$

6: **return** *x*

## 2 Methods

### 2.1 A multiscale Markov state model (mMSM)

**Figure 1.**
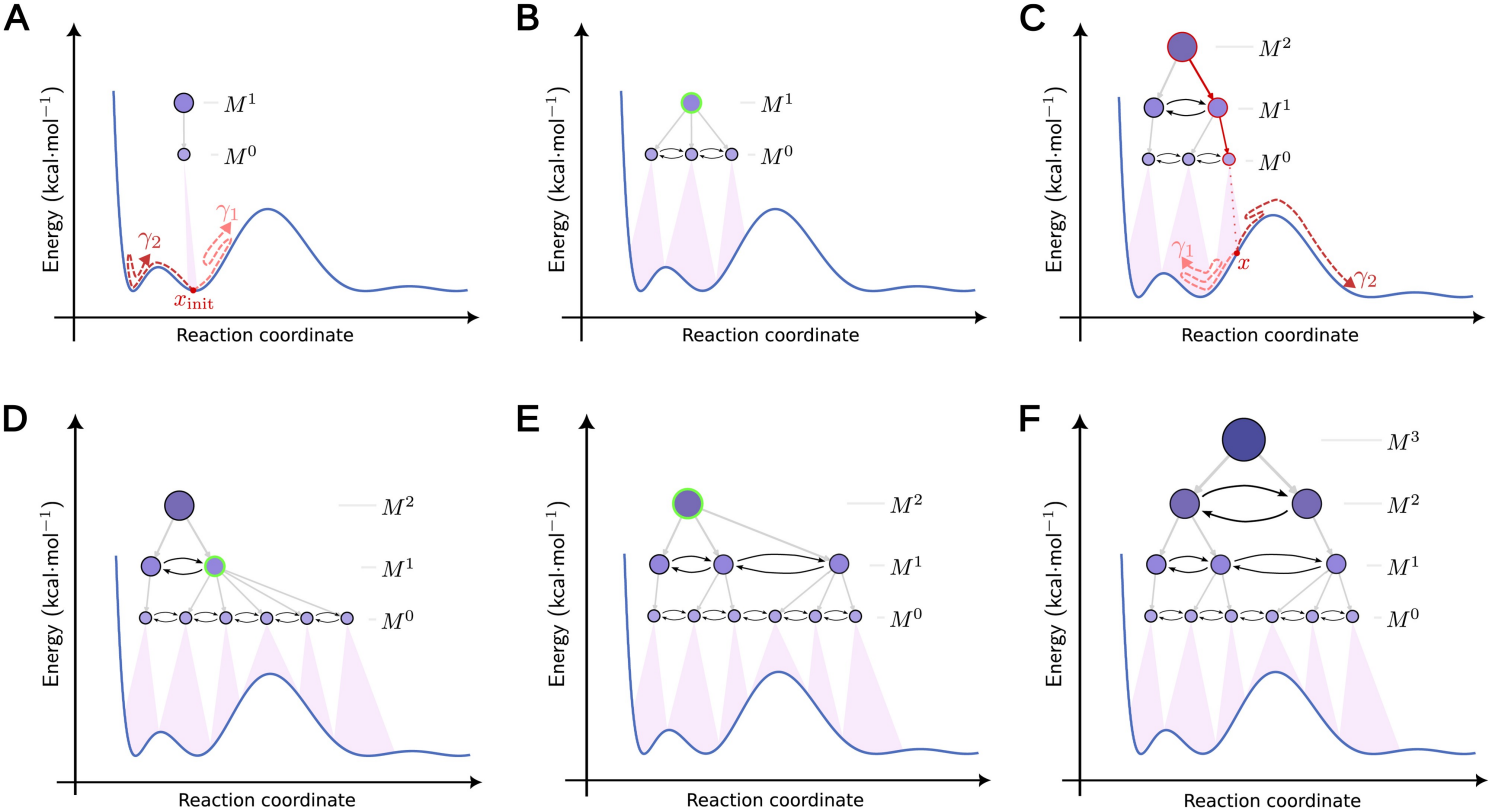
Illustration of mMSM-explore (Algorithm 1). (A) The mMSM is initialized using an initial configuration $x_{\mathrm{init}}$, from which one or more fine-grained simulation trajectories $\gamma_{i}$ (curved arrows) are generated using some standard simulator. (B) The mMSM is updated to reflect data from the new trajectories, partitioning and merging states at every scale in the hierarchy as needed. (C) Multiscale adaptive sampling is repeatedly used to select states for further expansion of the mMSM, starting at the root macrostate and recursively going down the hierarchy until reaching a microstate (Algorithm 2), from which additional trajectories are generated. (D-F) The mMSM is repeatedly updated with the new trajectories. The blue curve represents a hypothetical 1-D energy landscape. The microstates in $M^{0}$ form the fine-grained base of the hierarchy, followed by three progressively coarse-grained macrostates ($M^{1},\ldots ,M^{3}$). Directed edges in level $M^{h}$ represent the transition probabilities in the corresponding transition matrix $T^{h}$. Nodes highlighted in green denote macrostates selected for update (Algorithm S1, available as supplementary data at *Bioinformatics* online).

Intuitively, an mMSM is a hierarchy of progressively refined standard MSMs (MSMs; reviewed in Material A.1, available as supplementary data at *Bioinformatics* online). The MSM at the base of the hierarchy describes the system’s dynamics in terms of a finest-grained set of system states (Fig. 1). The union of these states covers $C_{\text{explored}}$, the currently explored portion of the system’s full configuration space C. Each subsequent MSM, higher in the hierarchy, is an aggregate description of its predecessor, that is, its states correspond to a partition of its predecessor’s states, thus providing an increasingly coarse-grained discretization of $C_{\text{explored}}$. This spatial hierarchy corresponds to a temporal hierarchy, where a base MSM at the bottom of the hierarchy is associated with a basic lag time $\tau_{0}$, and the MSM at level h is associated with a distinct lag time $\tau_{h}$. For simplicity, we can use $\tau_{h}=\kappa^{h}\tau_{0}$, where the factor κ>1 is a uniform temporal coarse-graining factor, chosen so that each MSM higher up in the hierarchy has a longer lag time relative to its predecessor, while remaining small enough to ensure gradual coarse graining (here, κ=2 by default). We refer to the states of the base MSM (h=0) as microstates, because of their fine granularity, and the states of all MSMs above it as macrostates. 

More formally, an mMSM is defined by two components: a hierarchical sequence of state sets with decreasing granularity, and a corresponding sequence of transition matrices associated with increasing lag times. The sequence of state sets is denoted by $\left(M^{0},\ldots ,M^{H}\right)$, where *H* is the number of levels in the MSM hierarchy. The first set $M^{0}=\{S_{1}^{0},S_{2}^{0},\ldots ,S_{n_{0}}^{0}\}$ is termed the base set, comprising $n_{0}$ disjoint microstates that jointly cover $C_{\text{explored}}$. $M^{h}=\{S_{1}^{h},\ldots ,S_{n_{h}}^{h}\}$ is a partition of $M^{h-1}$ to $n_{h}$ non-empty and disjoint subsets, or macrostates, where $n_{h}$ is a non-increasing sequence of integers. The last set $M^{H}$ contains a single macrostate $S_{1}^{H}$. This hierarchical partitioning can be represented using a tree structure where $S_{i}^{h+1}$ is the parent of $S_{j}^{h}$ if and only if $S_{i}^{h}\in S_{j}^{h+1}$, where the root is $S_{1}^{H}$ and the leaves are the microstates $\left\{S_{1}^{0},S_{2}^{0},\ldots ,S_{n_{0}}^{0}\right\}$. Each set of states $M^{h}\in \left(M^{0},\ldots ,M^{H}\right)$ is associated with a transition matrix $T^{h}\in \left(T^{0},\ldots ,T^{H}\right)$ among its states over a progressively longer lag time $\tau_{h}$. The Markov property implies that the transition probabilities are independent of past states given the present state:


$$T_{k,l}^{h}=Pr\left[S^{h}(t+\tau_{h})=S_{l}^{h}\;|\;S^{h}(t)=S_{k}^{h}\right]$$

where $S^{h}(t)$ and $S^{h}(t+\tau_{h})$ indicate the system’s states at time points *t* and $t+\tau_{h}$, respectively, within the set $M^{h}$ in the mMSM hierarchy.

### 2.2 mMSM-explore

**Figure 2.**
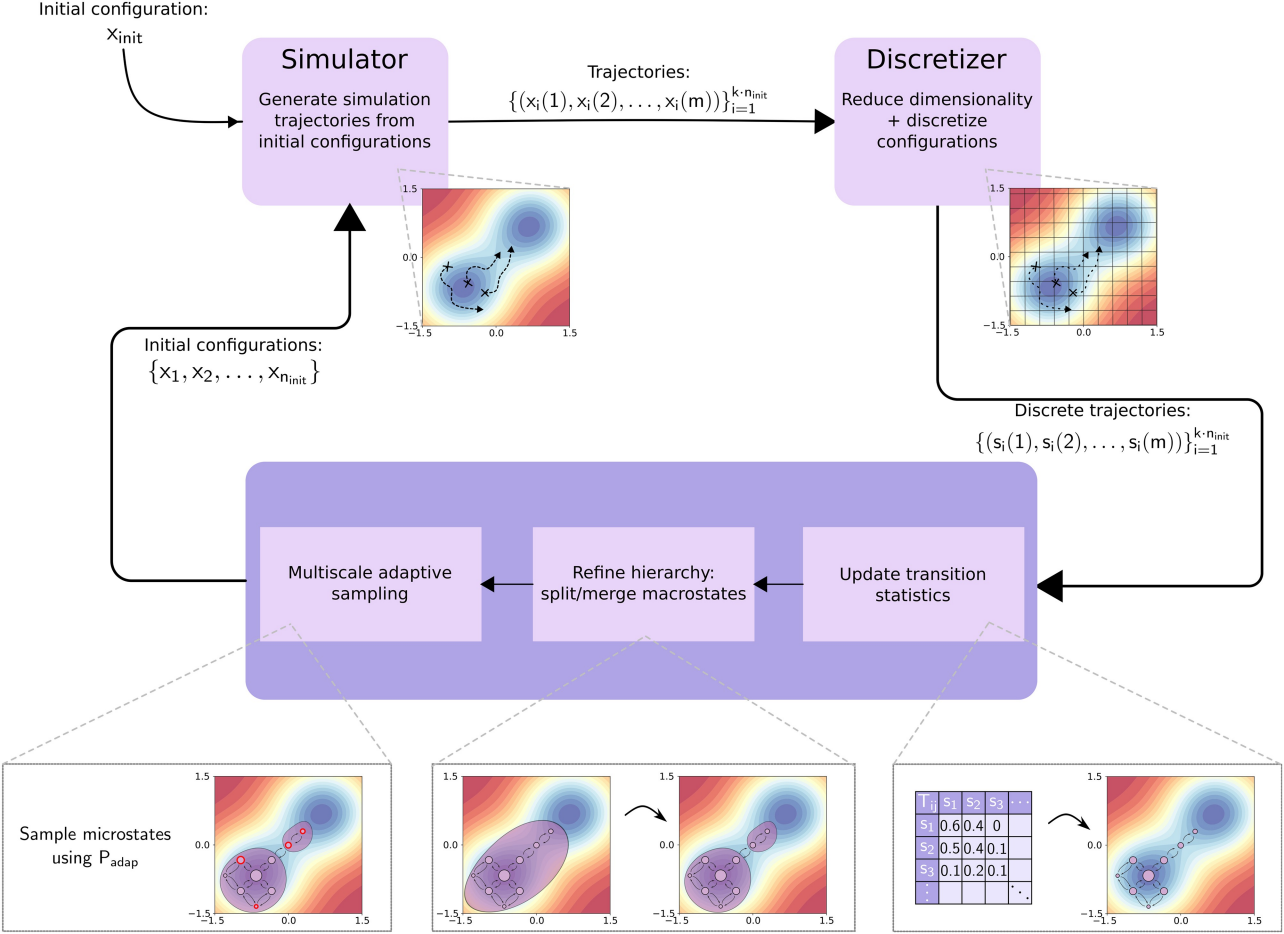
Overview of the mMSM-explore algorithm.

The mMSM-explore algorithm generates an mMSM data structure for a given dynamical system (Algorithm 1; Figs 1–2). Similarly to standard adaptive algorithms for constructing a conventional MSM (Husic and Pande 2018), it iteratively expands the mMSM data structure using data from one or more fine-grained simulation trajectories, which are generated by a standard simulator. Starting from an initial configuration $x_{\mathrm{init}}$, it simulates the system repeatedly (Fig. 1A), updates the existing mMSM (Fig. 1B), and adaptively utilizes the current mMSM for its own expansion by generating additional fine-grained trajectories and updating the mMSM hierarchy as needed (Fig. 1C-F). The process is then repeated until a satisfactory model is attained. However, unlike standard adaptive algorithms, mMSM-explore must employ a hierarchical update procedure to maintain the multiscale hierarchy. In addition, unlike standard adaptive sampling, it leverages the multiscale structure to more efficiently guide its own expansion.

#### 2.2.1 Updating the mMSM

The update procedure of mMSM-explore takes the set of recently simulated trajectories ${\left\{\gamma_{i}\right\}}_{i=1}^{k}$ as input, where each $\gamma_{i}$ comprises *m* consecutive microstates. These trajectories are first used to update the transition matrix $T^{0}$ of the base level ($M^{0}$), that is, to update transition statistics between the system’s microstates (Algorithm S1, lines 3–14; Equation S1, available as supplementary data at *Bioinformatics* online). This update is followed by bottom-up propagation of transition statistics through the hierarchy of progressively coarse-grained transition matrices (Fig. 1D–F; Algorithm S1, lines 16–19, available as supplementary data at *Bioinformatics* online). At each level of the hierarchy, the transition matrices $T^{1},\ldots ,T^{H}$ are recursively updated based on the aggregated transition probabilities of their children states (Equation S2, available as supplementary data at *Bioinformatics* online).

#### 2.2.2 Identifying temporal communities

To correctly capture the system’s multiscale temporal dynamics, the update procedure may merge neighboring macrostates or split them into finer partitions, after updating their transition statistics (Fig. S2, available as supplementary data at *Bioinformatics* online; Algorithm S1, lines 20–36, available as supplementary data at *Bioinformatics* online). Specifically, to refine the hierarchical structure, modularity-based graph clustering (Traag *et al.* 2019) is used to identify *temporal communities*, which are subsets of states with fast internal transitions relative to outward transitions, aligned with the notion of metastable states (Table 1).

**Table 1.** Glossary.

Symbol	Term	Meaning
$M^{h}$	set of states	The *h*th set of states in the mMSM hierarchy
$S_{i}^{0}$	microstate	The *i*th microstate in $M^{0}$, the lowest (finest-grained) set of states, which is obtained by discretizing the configuration space into a set of compact volumes
$S_{i}^{h}$	macrostate	The *i*th macrostate in $M^{h}$ for h≥1, corresponding to a union of some states in an underlying level $M^{h-1}$
$T^{h}$	transition matrix	Transition matrix specifying transition probabilities among the states of $M^{h}$
$\tau_{h}$	lag time	Timestep size associated with the transition matrix $T^{h}$ among the states of $M^{h}$
−	temporal community	A set of densely connected states

#### 2.2.3 Multiscale adaptive sampling

The multiscale adaptive sampling algorithm recursively samples a child macrostate, starting at the root macrostate, until a microstate is reached (Algorithm 2; Fig. S1, available as supplementary data at *Bioinformatics* online; Material A.3, available as supplementary data at *Bioinformatics* online). At each level of the hierarchy, it samples from some distribution $P_{\text{adap}}$ over its children, e.g. an exponential distribution inversely proportional to the number of visits to a given child, thus integrating information from every scale. Then, it selects a representative configuration from the chosen microstate, which serves as the initial configuration for subsequent simulations.

## 3 Results

### 3.1 Two spheres restrained by a multiscale spring potential

**Figure 3.**
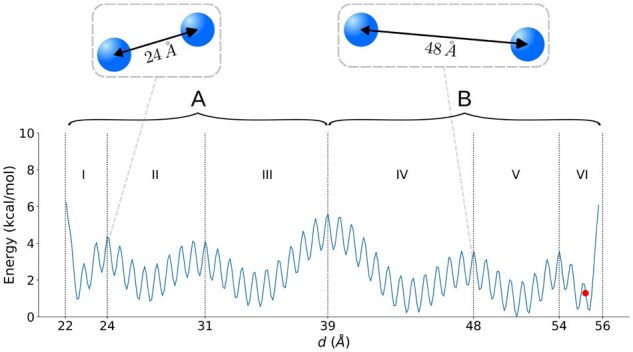
The free energy landscape for the 6-dimensional system of two spheres. The free energy is shown as a function of the distance between the sphere centers. It was computed from the distribution of states in a long naive simulation (40 000 ns) using a Boltzmann inversion with 0.1Å bins. The red point indicates the initial distance used for all simulations of this system. A and B indicate the two largest low-energy basins, separated by an energy barrier at d=39Å; roman numerals indicate six low-energy basins at an intermediate resolution.

We first used mMSM-explore to generate an mMSM for a simple toy system, consisting of two particles (spheres) of radius R=5Å, restrained to each other based on their distance. The distance-dependent restraint was designed to have a multiscale structure in the sense that it contains multiple nested energy minima (Fig. 3), serving as a template for real-world biomolecular systems, where multi-body interactions across many scales give rise to nested energy minima (Ayton *et al.* 2007). The system’s energy potential also includes an excluded volume restraint with a spring constant of $1.0\;\text{kcal}\cdot {\text{mol}}^{-1}\cdot {Å}^{-2}$. The system dynamics were simulated using Brownian dynamics, that is, overdamped Langevin dynamics (Schlick 2002). The diffusion coefficients for the spheres were assigned by assuming a Stokes radius of *R* for molecules diffusing in water (Berg and von Hippel 1985).

We initialized mMSM-explore from a configuration near the upper bound of the distance range (Fig. 3, red circle at d=55.25Å), using a temperature of 300 K and an integration timestep of 0.03 ps for the underlying MD simulation, for a total simulation time of 1000 ns, and a basic lag time of $\tau_{0}=0.03$ ps, where the implied timescales (relaxation times implied by the transition matrix) plateau for the five slowest processes in the system (Fig. S9A, available as supplementary data at *Bioinformatics* online). The individual configurations were discretized to microstates using K-centers clustering (Material A.4, available as supplementary data at *Bioinformatics* online) over the distance between the two spheres, with a cluster diameter of 0.05Å.

**Figure 4.**
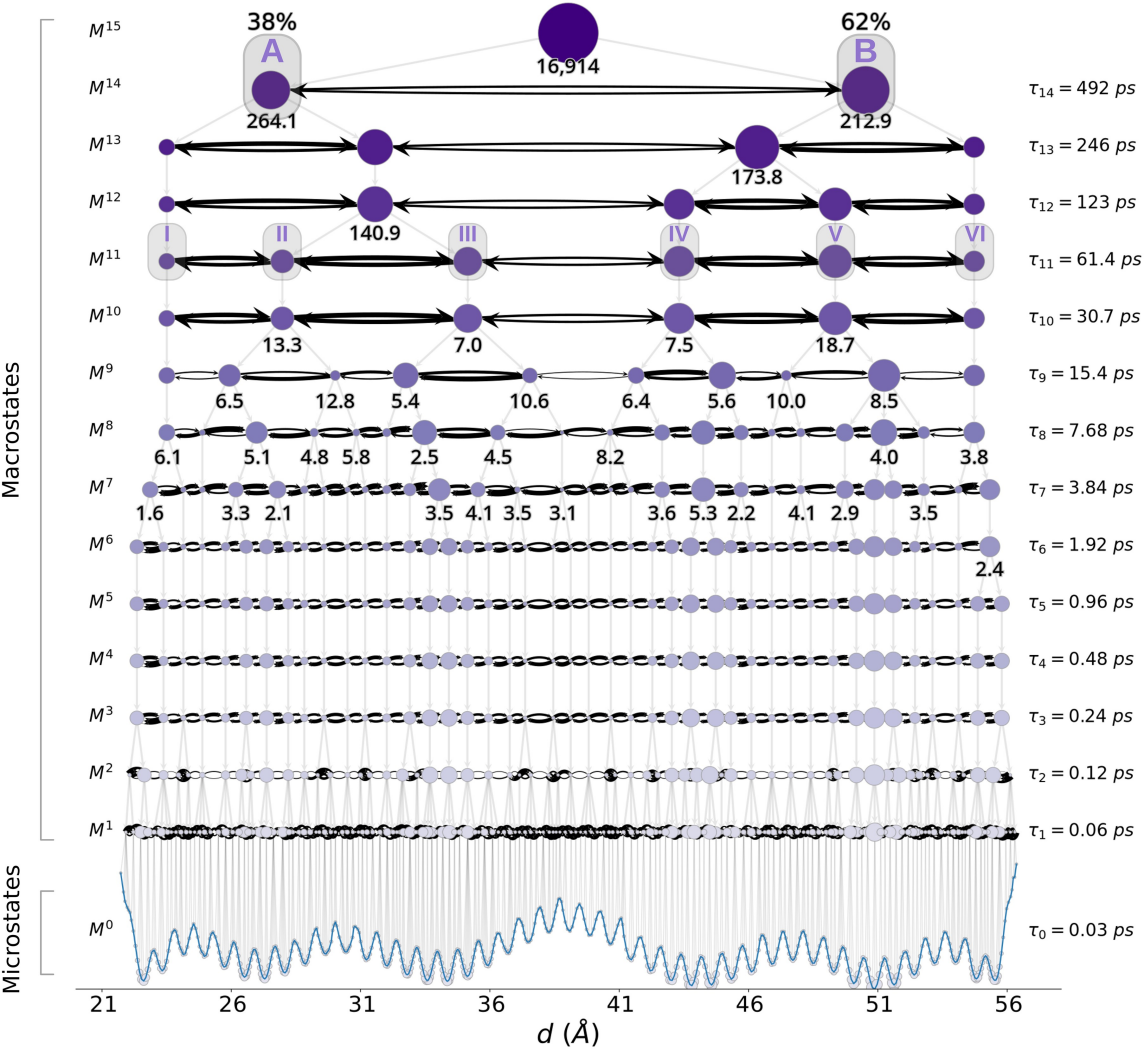
A representative output mMSM for the two-spheres system, generated using mMSM-explore. Each node corresponds to a system state, where nodes at higher levels in the hierarchy correspond to more coarse-grained system macrostates (darker purple color). Starting from the root macrostate (top), each macrostate is the union of its direct children, indicated by gray vertical or diagonal arrows. The leaves are the system’s microstates, plotted as light-purple nodes at the bottom along with the free energy function (blue curve). Designations of primary energy basins corresponding to those labeled in Fig. 3 are indicated as gray rounded rectangles at level $M^{11}$. The node sizes are proportional to the estimated stationary probabilities of the corresponding states. For the A and B energy basins, the probabilities are also stated numerically above their respective nodes. Horizontal arrows indicate transitions between states at the same level. For visual clarity, self-transitions are omitted. The widths of the horizontal edges are proportional to the transition probabilities between these states over $\tau_{0}=0.03$ ps. Numbers under a subset of the macrostates denote the relaxation time (in picoseconds) computed for the transitions between a macrostate’s children. The lag time associated with each level of granularity is indicated on the right.

In the resulting mMSM, the output macrostates correctly capture the low-energy basins across the various temporal scales (Fig. 4). For example, the children of the root macrostate (Fig. 4, level $M^{14}$) capture the two largest low-energy basins (Fig. 3, basins A and B). For each state at every scale, the mMSM also provides estimates for both the stationary probability of being at that macrostate, and the relaxation time for equilibrating among its children, where relaxation time is calculated through an eigenvector analysis of the transition matrix among these children (Prinz *et al.* 2011). For example, the inferred stationary probabilities to be in states A and B are 38% and 62%, respectively, and the estimated relaxation time for equilibrating between A and B equals 16 914 ps. The finer-grained macrostates of $M^{11}$ capture the six intermediate low-energy basins (Fig. 3, designated I to VI), with an estimated relaxation time on the order of 6.1 ps to 18.7 ps, significantly faster than between the main energy basins. At even lower (fine-grained) levels of the hierarchy (Fig. 4, $M^{6}$ to $M^{3}$), the macrostates capture the low-energy basins of the smallest scale. Finally, the macrostates in $M^{1}$ comprise subsets of microstates in $M^{0}$ with maximal granularity. This result was largely consistent in all 10 independent runs of mMSM-explore (Fig. S4, available as supplementary data at *Bioinformatics* online).

**Figure 5.**
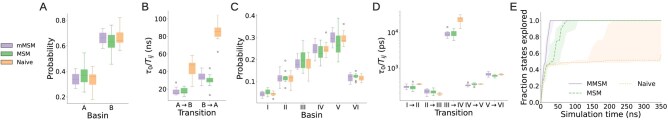
Comparison of accuracy and exploration rates using mMSM (purple; 10 independent runs of 1000 ns each), MSM (green; 10 independent runs of 1000 ns each), and naive simulation (orange; 10 independent runs of 20 000 ns each) for the two-spheres system (Material A.7, B.1, available as supplementary data at *Bioinformatics* online). (A–B) Estimated stationary probabilities and one-step transition times ($\tau_{0}/T_{ij}$) for energy basins A and B (Fig. 3). Median values are indicated within each box, which spans the first to third quartiles, with whiskers extending 1.5 times the interquartile range and empty circles indicating outliers. Naive-simulation estimates serve as the ground truth. (C, D) Same as panels A and B, for the six intermediate energy basins (Fig. 3). (E) Median fraction of visited states along the interval between d=22 Å and d=56 Å as a function of simulation time; shaded regions indicate 95% confidence intervals. See also Fig. S5, available as supplementary data at *Bioinformatics* online.

To evaluate the accuracy of the inferred stationary distributions and transition probabilities, we averaged multiple independent mMSM-explore runs to account for the system’s inherent stochasticity (Fig. 5, purple). As the ground truth, we computed these statistics over multiple long naive simulations (Fig. 5, orange). A similar analysis was performed for standard MSMs (Material A.7, available as supplementary data at *Bioinformatics* online) to provide a baseline for comparison (Fig. 5, green). We note that among these three methods, only mMSMs are capable of identifying low-energy basins in an unsupervised manner, without prior knowledge of their temporal scale. To facilitate the comparison, we therefore performed manual binning into the low-energy basins for the standard MSMs and naive simulations (Fig. 3).

For the two lowest-energy basins, the median stationary probabilities are broadly consistent with the ground truth values for both mMSMs and standard MSMs (Fig. 5A). On the other hand, the median one-step transition times, while capturing the overall timescales, are consistently underestimated by 61%-67% (Fig. 5B). However, because both the A→B and B→A rates are underestimated by similar factors, this bias largely cancels out when computing the stationary probabilities. We repeated this analysis for the six intermediate energy basins; the median stationary probabilities are also broadly consistent with the ground truth values (Fig. 5C). The estimates of the one-step transition times are also mostly consistent: transitions not crossing the main barrier (between basins III and IV) had median relative errors ranging from 0.5% to 21%, while the rates for the two transitions crossing the main barrier were underestimated by 59% to 66% (Fig. 5D; Fig. S5A, available as supplementary data at *Bioinformatics* online), likely explaining the error for the A–B transitions up the hierarchy. Throughout this analysis, the observed errors are similar to those present in the baseline estimates produced by a standard MSM, suggesting that the discrepancies are likely due to local non-Markovian behavior, and not a result of bias introduced by the multiscale sampling process (Material A.5, available as supplementary data at *Bioinformatics* online).

We next monitored the speed with which mMSM-explore visits new system states compared with either a standard MSM or a naive simulation used as a reference (Fig. 5E; Fig. S5B–D, available as supplementary data at *Bioinformatics* online). The median mMSM-explore run visited all feasible system states after 27 ns of total simulation time (Fig. 5E; 95% CI 22–31 ns). In contrast, the median standard MSM run took 74 ns to visit all states, with a larger spread than for mMSM-explore (Fig. 5E; 95% CI 63–101 ns). Finally, the median naive simulation visited all states only after 457 ns, with an even larger spread (Fig. 5E; 95% CI 210–975 ns). Thus, for the two-spheres system, mMSM-explore constructs a more elaborate data structure in half the time compared with a standard MSM, and it does so seven times faster compared with a naive simulation.

### 3.2 Alanine dipeptide

#### 3.2.1 Alanine dipeptide discretized based on the ϕ,ψ angles

**Figure 6.**
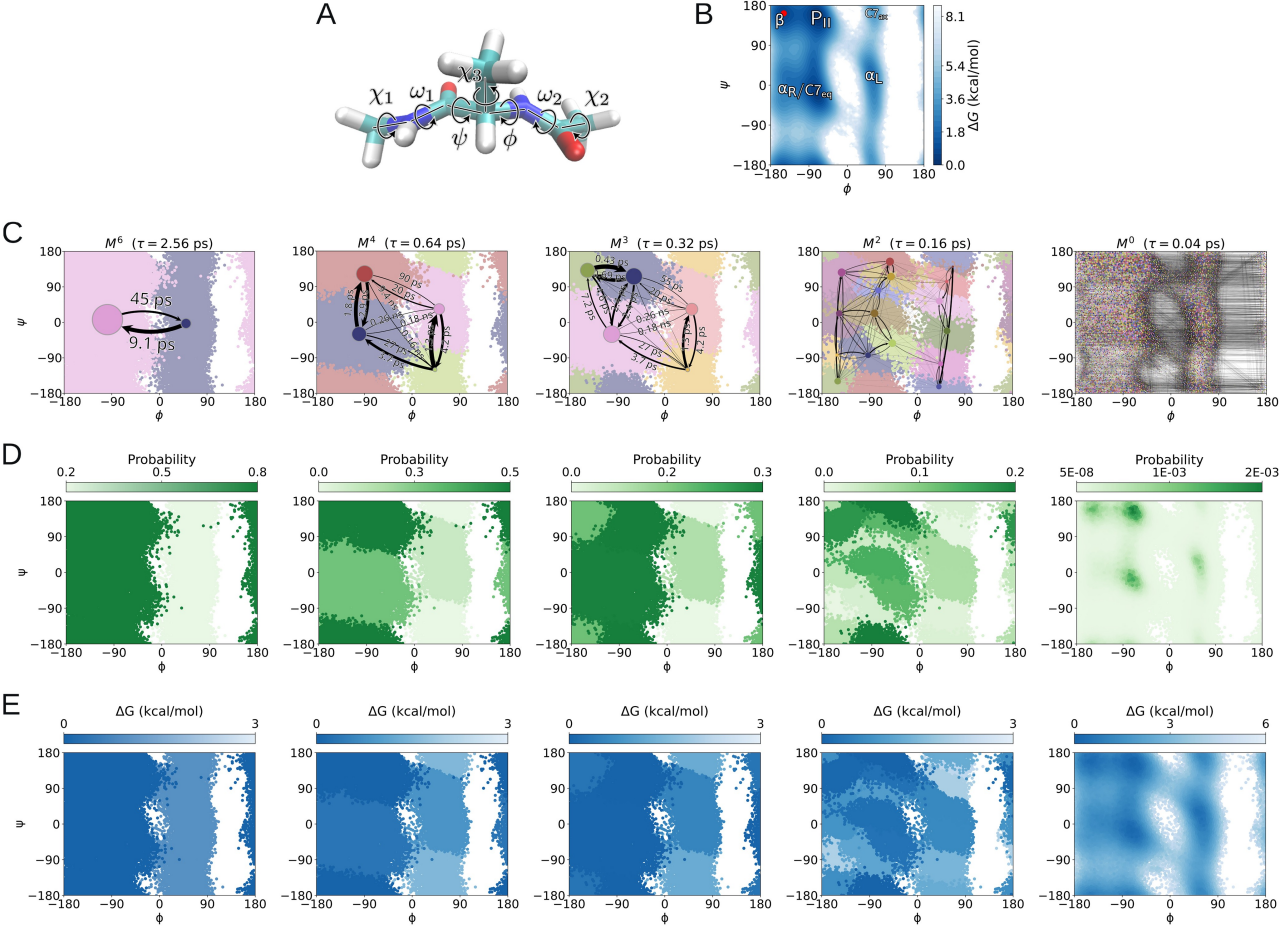
A representative output mMSM for alanine dipeptide. (A) A stick representation of an alanine dipeptide molecule with its seven torsion (dihedral) angles indicated. The atoms defining each angle are detailed in Table S1, available as supplementary data at *Bioinformatics* online. (B) The free energy landscape of alanine dipeptide mapped across the two reaction coordinates ϕ and ψ, derived from a long naive simulation. The five known low-energy basins (Strodel and Wales 2008) are indicated over the corresponding regions in the plot: β, P${}_{\text{II}}$, $\alpha_{R}$/C7${}_{\text{eq}}$, $\alpha_{L}$ and C7${}_{\text{ax}}$. The red dot marks the initial configuration in all simulation runs. (C) MSMs in five levels ($M^{0}$, $M^{2}$, $M^{3}$, $M^{4}$ and $M^{6}$) of a representative 7-level mMSM hierarchy describing the dynamics of an alanine dipeptide molecule. The node sizes are proportional to the estimated stationary probabilities of the corresponding states. Edges are weighted proportionally to the transition probabilities between states over the basic lag time $\tau_{0}=0.04$ ps; edge labels denote $\tau_{0}/T_{ij}$, the one-step transition times at lag $\tau_{0}$. (D) The estimated stationary probabilities for the states in each of the five levels. (E) Estimated free energies for the states in each of the five levels.

In the second benchmark, we constructed an mMSM data structure for an alanine dipeptide molecule, based on Langevin dynamics simulations in implicit solvent. The thermodynamic and kinetic properties of alanine dipeptide have been studied extensively, both experimentally and *in silico* (Gfeller *et al.* 2007, Feig 2008, Strodel and Wales 2008, Parchanšký *et al.* 2013). It comprises 22 atoms (66 degrees of freedom including translation and rotation), but it largely operates along a two-dimensional manifold (reaction coordinates), corresponding to the molecule’s torsion (dihedral) angles ϕ and ψ (Fig. 6A–B), transitioning between five well-characterized low-energy basins (Fig. 6B) (Strodel and Wales 2008).

We performed 10 independent runs of mMSM-explore, all starting from a specific configuration within the β conformation (Fig. 6B), corresponding to $\phi =-149.6^{{}^{\circ}}$ and $\psi =158.3^{{}^{\circ}}$ Simulation was conducted at a temperature of 400 K, using an integration timestep of 2 fs, for a total length of 300 ns for each run. The basic lag time, $\tau_{0}$, was set to 0.04 ps, for which the implied timescales plateau for the three slowest processes in the system (Fig. S9B, available as supplementary data at *Bioinformatics* online). Configurations were discretized into microstates using K-centers in the ϕ,ψ space, using a cluster diameter of $6^{{}^{\circ}}$.

**Figure 7.**
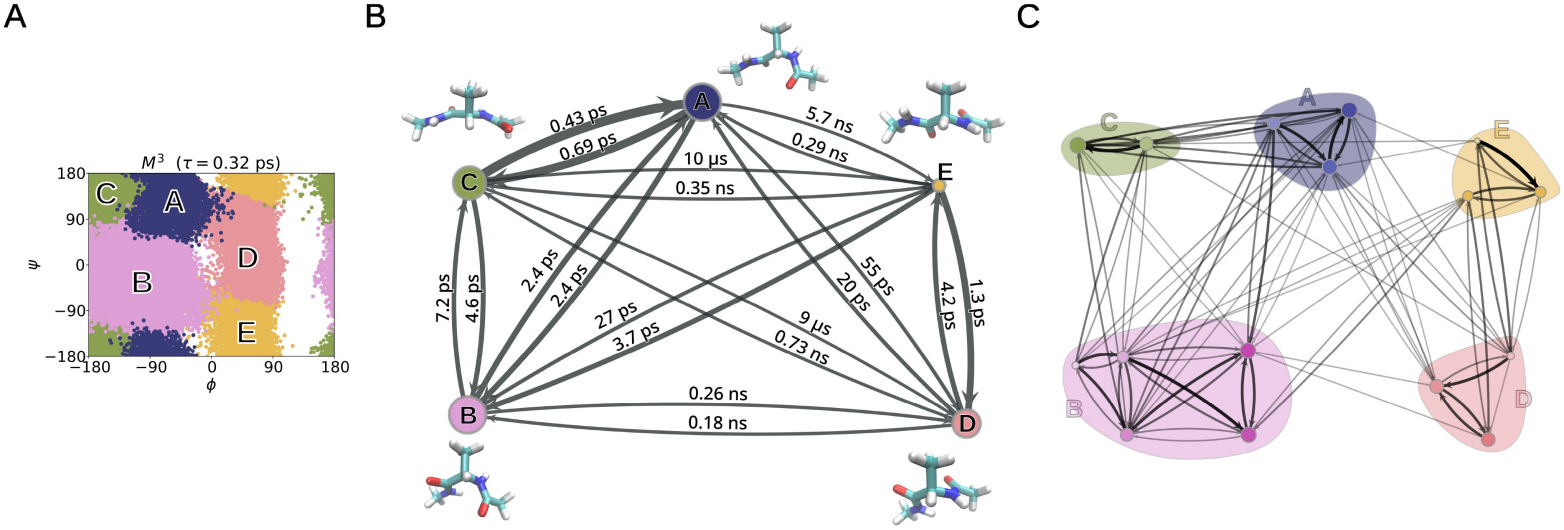
Identification of known molecular states of alanine dipeptide by mMSM-explore. (A) The partition at level $M^{3}$ from the representative mMSM. This partition corresponds to the five recognized low-energy basins (Fig. 6B): P${}_{\text{II}}$ (macrostate A), $\alpha_{R}$/C7${}_{\text{eq}}$ (macrostate B), β (macrostate C), $\alpha_{L}$ (macrostate D) and C7${}_{\text{ax}}$ (macrostate E). (B) Graphical representation of level $M^{3}$. The node sizes are proportional to the estimated stationary probabilities of the correspionding macrosctates. Edges indicate transitions between macrostates. For visual clarity, self-transitions are omitted. Edge widths are proportional to the transition probabilities between these macrostates at the basic lag time $\tau_{0}=0.04$ ps. The one-step transition time between the macrostates is indicated near the corresponding edge. Adjacent to each macrostate is an illustration of a representative conformation from that macrostate. (C) Graphical representation of level $M^{2}$ of the representative, illustrated on top of the coarser partition from level $M^{3}$. The colors and letters indicate each of the five macrostates of $M^{3}$.

The mMSM generated by mMSM-explore accurately captures the topology of the two-dimensional energy landscape, including its known low-energy basins (Fig. 6C–E). In a randomly-selected representative mMSM, the root macrostate has two children states (level $M^{6}$). The first state, corresponding to the main region of configurations containing the known P${}_{\text{II}}$, $\alpha_{R}$/C7${}_{\text{eq}}$ and β (Fig. 6B, left side) basins; and a low-probability region containing the known $\alpha_{L}$ and C7${}_{\text{ax}}$ basins (Fig. 6B, right side). Lower levels down the hierarchy provide finer partitions of $M^{6}$. The partition at $M^{3}$ is of particular interest, as its macrostates correspond to the five known states of the molecule (Fig. 7A; cf. Fig. 6B). Thus, the mMSM recovers the known hierarchy of macrostates for alanine dipeptide across scales, without requiring prior knowledge of the temporal scale associated with each level. At even lower levels, the hierarchy reveals more detailed partitions of these macrostates. Consequently, the multiscale structure enables detailed inspection of their conformational dynamics, facilitating the identification of transition states between them (Fig. 7C).

As a reference for model evaluation, we generated standard MSMs using 10 independent runs (Material A.7, available as supplementary data at *Bioinformatics* online). The settings for each run were identical to the mMSM-explore runs, including a total simulation time of 300 ns per run. In addition, we ran 10 independent naive simulation runs, each running for 1000 ns (Material B.2, available as supplementary data at *Bioinformatics* online).

**Figure 8.**
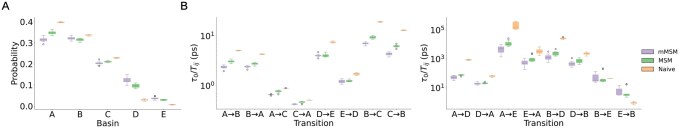
Comparison of accuracy and exploration rates using mMSM (purple; 10 independent runs of 300 ns each), MSM (green; 10 independent runs of 300 ns each), and naive simulation (orange; 10 independent runs of 1000 ns each) for the alanine dipeptide system. (A) Estimated stationary probabilities for the macrostates of level $M^{3}$ corresponding to the five known low-energy basins (Fig. 7A). Median values are indicated within each box, which spans the first to third quartiles, with whiskers extending 1.5 times the interquartile range. Naive-simulation estimates serve as the ground truth. (B) One-step transition time estimates for the same macrostates, split to fast (left) and slow (right) transitions for visual clarity.

We first compared the models’ estimates for select stationary probabilities and one-step transition times, focusing on the five known low-energy basins (Fig. 6B), using the exact partition described in Fig. 7A to assign each (ϕ,ψ) region to one of the five corresponding macrostates. In both the mMSMs and the MSMs, the estimated stationary probabilities are broadly consistent with the ground truth, but they are slightly underestimated for the high-probability macrostates (A, B, C), and slightly overestimated for the low-probability ones (D, E) (Fig. 8A). The estimated one-step transition times between macrostates are mostly ranked consistently with the ground truth (Fig. 8B). However, they are underestimated by 17% to 96% (and approximately 290% for a single transition) in both the mMSM and in the MSM compared to a naive simulation, possibly due to some local non-Markovian behavior, as the implied timescales are not fully convergent for the fourth and fifth slowest processes in the system (Fig. S9B, available as supplementary data at *Bioinformatics* online).

**Figure 9.**
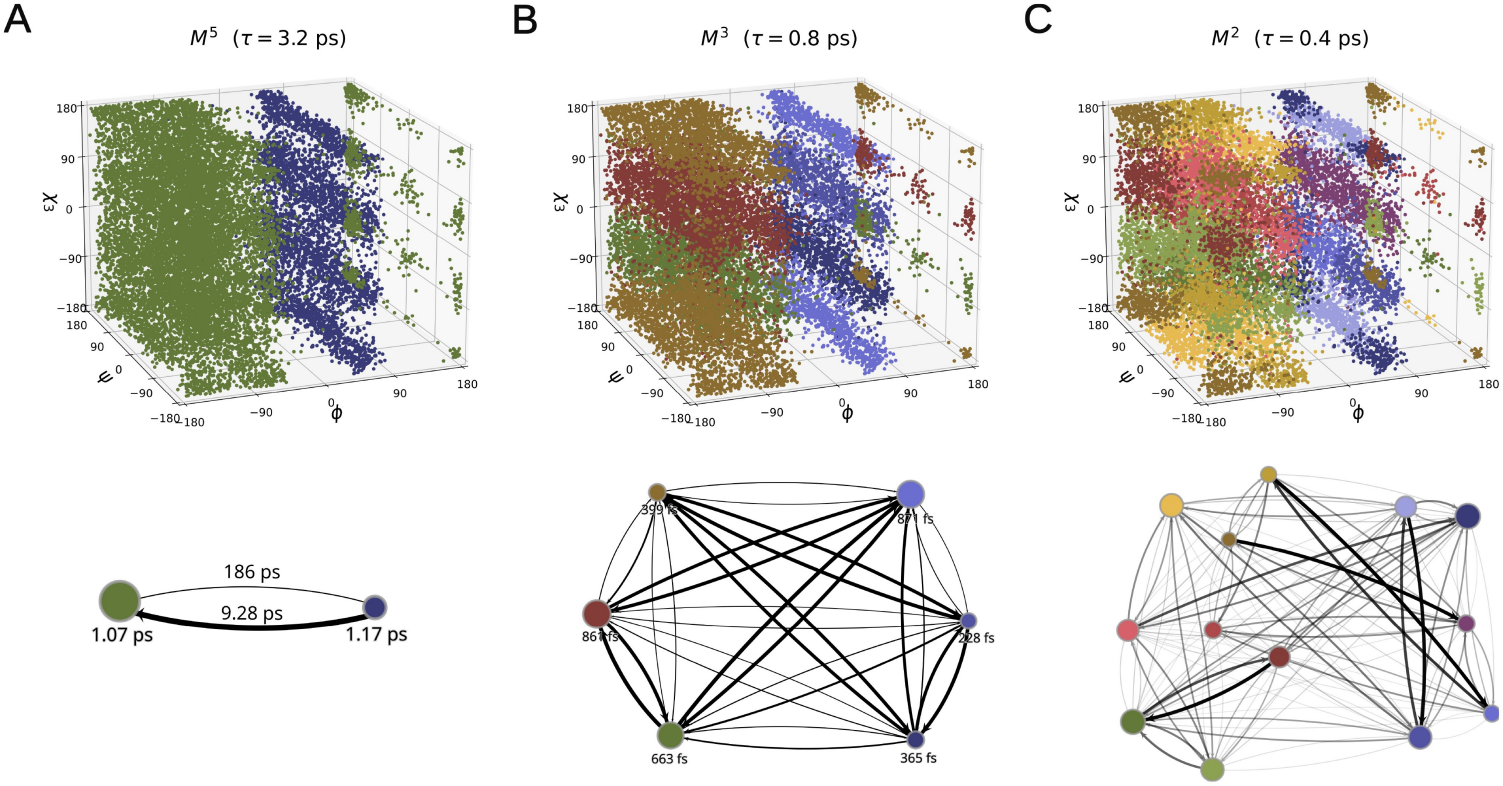
Visualization of the representative mMSM, showing macrostate partitions at levels $M^{5}$ (A), $M^{3}$ (B) and $M^{2}$ (C). In each panel, the top part plots the projection of microstates onto the ϕ, ψ, and $\chi_{3}$ reaction coordinates; colors indicate the partition to macrostates at the respective level. The bottom part of each panel depicts the corresponding transition network, where nodes represent macrostates, colored as in the top plots and scaled according to their stationary probabilities. Edges indicate transitions, with widths proportional to the transition probabilities over the basic time lag $\tau_{0}=0.1$ ps, with labels denoting the one-step transition times. Self-transitions are omitted for visual clarity. Labels beneath the nodes represent local relaxation times. For readability, some labels are omitted for (B) and (C). In (C), edge transparency is proportional to the transition probabilities, in addition to edge width. See also Fig. S7, available as supplementary data at *Bioinformatics* online.

When comparing the efficiency of discovering new states (Fig. S6, available as supplementary data at *Bioinformatics* online), mMSM-explore performs as efficiently as standard adaptive sampling for generating a standard MSM, visiting 80% of all (ϕ,ψ) configuration states after a median simulation time of 85 ns and 140 ns, respectively. Both methods are considerably more efficient than a naive simulation, where 80% of states are visited after 395 ns, eventually covering 83% of states only after its full 1000 ns run.

#### 3.2.2 Alanine dipeptide discretized based on all seven torsion angles

For the third benchmark, we evaluated mMSM-explore’s effectiveness at identifying the main degrees of freedom governing system dynamics from an extended set of candidate features. For this purpose, we constructed the mMSM for alanine dipeptide using the same simulation setup as in Section 3.2.1, but discretized the full configuration space into microstates based on all seven torsion angles of the alanine dipeptide molecule (Fig. 6A), using K-centers with a cluster diameter of $120^{{}^{\circ}}$. The basic lag time, $\tau_{0}$, was set to 0.1 ps.

The mMSM structure generated by mMSM-explore accurately identifies the primary degrees of freedom, ϕ and ψ, from the set of candidate features (Fig. 9). Moreover, the macrostates composing the hierarchical structure align precisely with regions in the ϕ and ψ space corresponding to known low-energy basins, without requiring prior knowledge of neither the particular reaction coordinates by which they are defined, nor the relevant timescales. In addition, the output mMSM highlights a possible role for the $\chi_{3}$ torsion angle in modulating the transition rates between the known low-energy basins.

Specifically, at the top level ($M^{5}$), which represents the system’s slowest dynamical process, the partition consists of two macrostates. Projection of the microstates associated with these two macrostates onto the $\phi ,\psi ,\chi_{3}$ coordinates reveals two distinct regions corresponding to the known low-energy basins depicted in Fig. 6C ($M^{6}$). The first macrostate (left) encompasses the β, P${}_{\text{II}}$, and $\alpha_{R}$/C7${}_{\text{eq}}$ basins, while the second macrostate (right) includes the $\alpha_{L}$ and C7${}_{\text{ax}}$ basins (Fig. 9A). At the level below ($M^{4}$), these two macrostates are further subdivided along the $\chi_{3}$ coordinate, isolating a metastable state within the $\left[-90^{{}^{\circ}},90^{{}^{\circ}}\right]$ interval (Fig. S7B, available as supplementary data at *Bioinformatics* online). At level $M^{3}$, this metastable region is partitioned again, dividing it at the midpoint of the interval ($\chi_{3}=0^{{}^{\circ}}$) (Fig. 9B). Finally, at level $M^{2}$, the macrostates are partitioned along the ϕ,ψ plane, aligning with the five known low-energy basins shown in Fig. 7A (Fig. 9C). Interestingly, distinct transition probabilities are observed for states differing in their $\chi_{3}$ values (Fig. 9B-C), suggesting that $\chi_{3}$ plays a role in modulating transitions between the two primary macrostates at $M^{5}$ and among the five conformations identified at $M^{2}$.

We also evaluated the accuracy of the resulting representation by examining thermodynamic and kinetic quantities for the five known low-energy basins ϕ. The resulting estimates were broadly consistent with reference values derived from the naive simulations (Section 3.2.1 ϕ,ψ); detailed error statistics are provided in Fig. S8, available as supplementary data at *Bioinformatics* online.

### 3.2.3 Villin headpiece (HP35)

Finally, we applied mMSM-explore to characterize the hierarchical folding process of the 35-residue three-helix headpiece subdomain (HP35) of chicken villin, a widely used benchmark for fast-folding proteins (Ensign *et al.* 2007, Freddolino and Schulten 2009, Beauchamp *et al.* 2011, Nagel *et al.* 2023). As input, we used the precomputed 300 µs MD trajectory of the Lys24Nle/Lys29Nle mutant (Piana *et al.* 2012, Nagel *et al.* 2023). Each trajectory frame was represented by the previously defined 42 minimum heavy-atom distances corresponding to native contacts; the trajectories were then pre-processed and streamed to the mMSM-explore algorithm in sequential chunks (Material B.4, available as supplementary data at *Bioinformatics* online).

**Figure 10.**
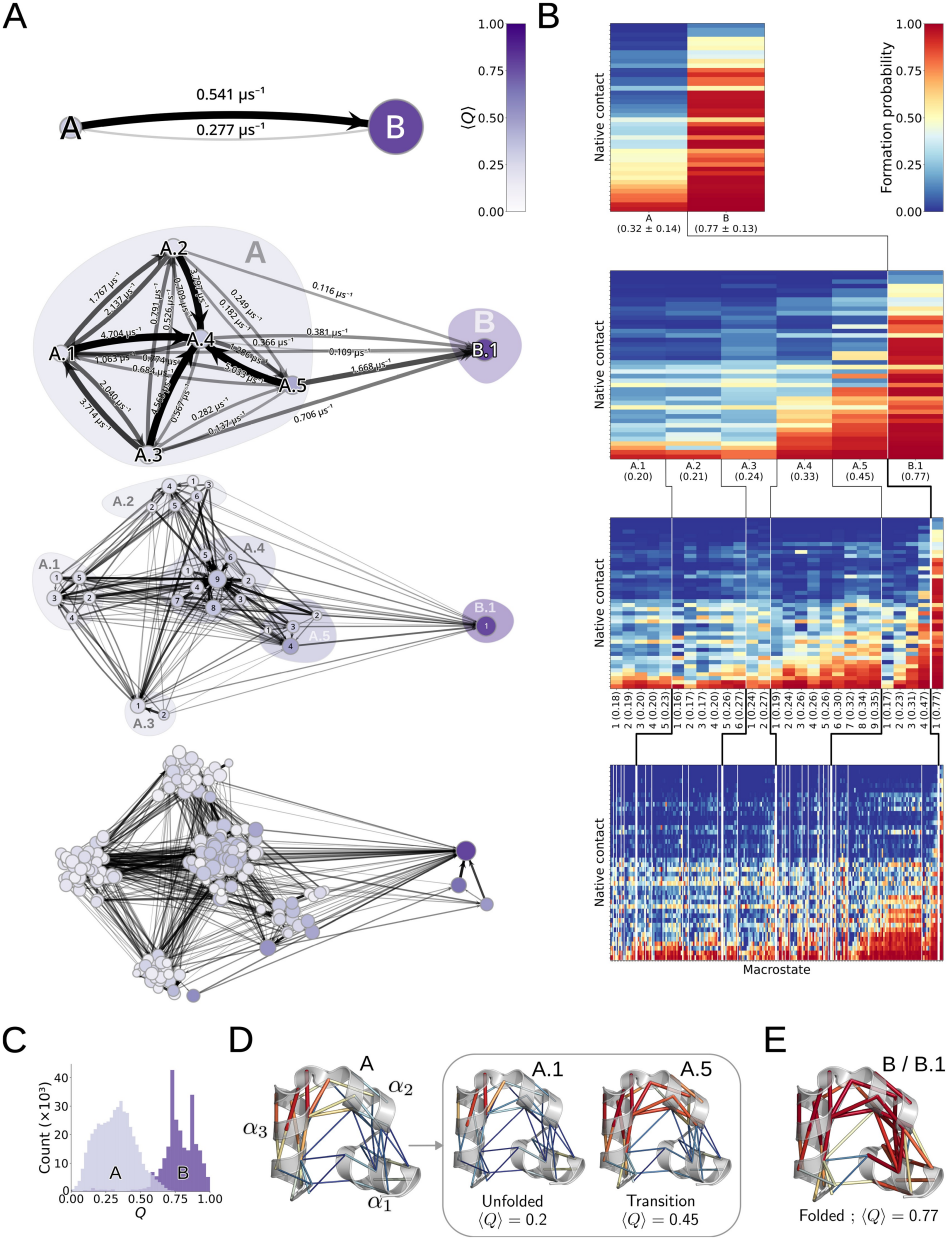
The output mMSM for the Villin headpiece (HP35). (A) mMSM hierarchy levels with increasing granularity (rows, top to bottom; microstates omitted); node sizes are proportional to the stationary probability of the corresponding macrostates, node colors encode mean fractions of native contacts 〈Q〉 (Nagel et al. 2023). Edge widths and opacities are proportional to the transition probability over a lag time of 100 ns. Edge labels, where present, indicate rates. For visual clarity, low-probability edges are omitted (thresholds: second row 0.01, third row 0.02, fourth row 0.04). (B) Heatmaps highlighting contact formation probability (color bar; contact threshold <4.5 Å) for the native contacts (y-axis) within each macrostate (x-axis) at each level in the mMSM hierarchy. The contacts are sorted by their mean prevalence throughout the simulation (Fig. S12, available as supplementary data at *Bioinformatics* online). The value of 〈Q〉 is indicated in parentheses for each macrostate (standard deviations are indicated in parentheses for the topmost level). (C) Distributions of Q for the two top-level macrostates A and B, corresponding to the unfolded and folded ensembles, respectively. (D, E) Structure-based visualization of the 42 native contacts and their probabilities for macrostates A and B, corresponding to the unfolded and folded ensembles, respectively. Down the hierarchy, the unfolded macrostate is resolved to finer-grained macrostates, such as those corresponding to the fully unfolded (A.1) and transition (A.5) ensembles. Edge widths are proportional to the formation probability within the macrostate, and edge colors are in accordance with Fig. 10B.

The generated mMSM hierarchy identifies distinct unfolded, intermediate, and folded macrostates, which emerge down the hierarchy (Fig. 10). We quantified native-state similarity using the mean fraction of formed native contacts 〈Q〉 (Nagel *et al.* 2023). At the top level of the mMSM hierarchy, the partition comprises two macrostates (Fig. 10A–B, first row), a low-*Q* population corresponding to an ensemble of predominantly unfolded conformations (〈Q〉≈0.32) and a high-*Q* population corresponding to an ensemble of predominantly folded conformations (〈Q〉≈0.77). The configurations in each of these topmost macrostates span a range of *Q* values (Fig. 10C), with little overlap at intermediate *Q* values. At the next level in the mMSM hierarchy, the folded macrostate remains stable, whereas the unfolded macrostate resolves into several lower-level macrostates (Fig. 10A–B, second row). A dominant transition macrostate (A.5, 〈Q〉≈0.45) has the highest probability to transition to the folded macrostate (B.1). A second, mid-transition macrostate with a lower 〈Q〉 (A.4, 〈Q〉≈0.33) also participates in the pathway. Contact heatmaps across these states reveal a sequential formation of native contacts: initial formation within A.1/A.2/A.3, followed by A.4, then A.5, and finally B.1 (Fig. 10B, second row). Consistent with a previous analysis of this trajectory (Nagel *et al.* 2023), contacts are first formed within helix $\alpha_{3}$ (A.1/A.2/A.3), then between helices $\alpha_{2}$ and $\alpha_{3}$ (A.4/A.5), and finally, between the two other pairs of helices (B.1) (Fig. 10D–E; Figs S12 and S13, available as supplementary data at *Bioinformatics* online). Down the hierarchy, the various unfolded and intermediate macrostates resolve into fine-grained macrostates, capturing their internal dynamics across a range of *Q* values (Fig. 10A–B, third and fourth rows). At this point, the folded *B* basin also partitions for the first time, with three fine-grained macrostates differing in their native-contact patterns and exhibiting distinct mean *Q* values, also revealing an intermediate macrostate where the contacts between helices $\alpha_{1}$ and $\alpha_{2}$ (B.1) have formed, but those between helices $\alpha_{1}$ and $\alpha_{3}$ have not (Fig. 10B, fourth row; Fig. S14, available as supplementary data at *Bioinformatics* online). Thus, mMSMs capture intricate folding dynamics across a hierarchy of scales without committing to a single level of granularity.

## 4 Discussion

In this work, we introduce the mMSM data structure for capturing system dynamics across multiple temporal scales, and mMSM-explore, an algorithm for generating an mMSM. This hierarchical perspective provides insight into the interplay between faster local fluctuations, intermediate motions, and slower global reorganizations, which could be overlooked by single-scale analyses. For example, in our analysis of alanine dipeptide in a seven-dimensional space, we identified the $\chi_{3}$ torsion angle as playing an intermediate role in regulating transitions previously observed in the slower ϕ and ψ coordinates (Fig. 9). For HP35, this multiscale view captures the usual unfolded and folded ensembles, reveals the partitioning of the unfolded state to fully unfolded and intermediate sub-states, and further resolves them into multiple states with differing native-contact patterns, providing a more detailed picture of the folding process without requiring prior knowledge of the relevant timescales (Fig. S10, Figs S13 and S14, available as supplementary data at *Bioinformatics* online). Due to its multiscale nature, this approach can be adapted straightforwardly for precise identification of transition states across temporal scales, via repeated algorithm runs or controlled perturbations (Dongen 2000, Gfeller *et al.* 2007).

To efficiently explore the accessible system states, we also introduced multiscale adaptive sampling, leveraging mMSM’s hierarchical representation for its own expansion, starting from the most coarse-grained macrostates at the top of the hierarchy down to the fine-grained microstates at its bottom (Algorithm 2; Fig. 1C). Our results indicate that mMSM-explore with adaptive sampling requires either the same or less computational effort compared to standard non-multiscale adaptive sampling, despite generating an elaborate multiscale representation in an unsupervised manner (Fig. 5E, Figs S5 and S6, available as supplementary data at *Bioinformatics* online).

In principle, an mMSM can be generalized to describe any dynamical system, and is thus applicable to a wide range of processes beyond molecular dynamics. For instance, an iterative optimization process for model parameters, such as training a neural network, can be viewed as a dynamic traversal of a surface in parameter space, where the surface is defined by the objective function. Although the parameter space of modern neural networks is vast, it can be projected onto lower dimensions to visualize this surface, known as the loss landscape (Li *et al.* 2018). In this context, nested energy minima are particularly important, as they can impede optimization by trapping the process in local minima, thereby degrading network predictive power. Other systems where mMSMs could be useful include MCMC samplers (Hoffman *et al.* 2014), denoising diffusion probabilistic models (Ho *et al.* 2020), and more. Applying mMSM-explore in these settings will only require the selection of an appropriate set of degrees of freedom for each task, a discretization scheme along these degrees of freedom, and a function for generating a series of successive system configurations from a specific initial configuration. In systems characterized by a hierarchical structure of nested energy minima, mMSM-explore is expected to accelerate exploration and provide a more thorough and informative representation of their dynamics, complementing approaches for accelerating simulations by predicting future time steps across temporal scales (Mintz and Raveh 2025).

We conclude by addressing some limitations of mMSMs and how to resolve them. Because mMSMs build directly on the methodology of standard MSMs, they inherit many of the same challenges but also benefit from a large body of established solutions. For example, existing methods for reaction-coordinate selection, lag-time validation, and dimensionality reduction in the context of standard MSMs (Pérez-Hernández *et al.* 2013) are all directly applicable to mMSMs. Furthermore, due to the hierarchical exploration of the system’s states, mMSM-explore may also identify underlying reaction coordinates from a broad candidate set (Section 3.2.2; Fig. 9). At the same time, as in standard MSMs, discretization choices applied in the context of very large biological systems can yield exceptionally large numbers of states. Beyond the issue of sparsity in transition counts, this proliferation of states can induce a deep and wide multiscale hierarchy; maintaining and updating the associated mMSM can therefore introduce noticeable computational complexity. Another limitation of mMSMs is conceptual: our identification of macrostates at a given timescale currently relies on modularity-based community detection, but it does not, by itself, constitute a formal test of metastability, thus motivating the development of scale-aware diagnostics for metastability. For example, existing diagnostics such as local validation of implied timescales consistency (Prinz *et al.* 2011), spectral-gap criteria (Sarich *et al.* 2010), or Chapman–Kolmogorov validation (Prinz *et al.* 2011), can be extended to test a multiscale hierarchy of macrostates. Variational and deep learning approaches for identifying system states (Noé and Nuske 2013, Mardt *et al.* 2018, Sidky *et al.* 2019) can also be adapted to optimize the identification of temporal communities across scales. Finally, the practical speed of mMSM-explore can be accelerated in several ways. Our multiscale adaptive sampling approach can be made more computationally efficient by transforming existing techniques used for standard adaptive sampling (Zimmerman and Bowman 2015, Hruska *et al.* 2018, Strahan *et al.* 2023) to a multiscale setting. In addition, we will extend the methodological and software toolbox around mMSMs, also improving code design, software engineering, and distributed implementations, to significantly accelerate practical implementations. Looking ahead, the multiscale view provided by mMSMs will uncover how dynamic processes at different scales come together to facilitate the function of complex biological machines.

## Supplementary Material

btaf585_Supplementary_Data

## Data Availability

The code for reproducing the molecular dynamics simulations and all analysis steps, including detailed tutorials, are available in a public GitHub repository (https://github.com/ravehlab/mMSM).
