# Supplementary material for: Building multiscale Markov state models by systematic mapping of temporal communities
Source: Bioinformatics. 2025 Nov 25;42(1):btaf585. doi: 10.1093/bioinformatics/btaf585 (PMC12797069; doi:10.1093/bioinformatics/btaf585)
Supplement: btaf585_Supplementary_Data [file btaf585_supplementary_data.pdf]

Building multiscale Markov state models by systematic mapping of temporal communities: Supplementary material

Contents

A Supplementary methods **2**
A.1 A standard Markov State Model (MSM) . . . 2
A.2 mMSM-explore . . . 2
A.2.1 Estimation of transition model . . . 2
A.2.2 Partitioning to temporal communities . . . 3
A.2.3 Implementation details . . . 4
A.3 Multiscale adaptive sampling . . . 4
A.4 Discretization scheme . . . 6
A.5 Discretization error . . . 6
A.6 Additional procedures . . . 6
A.7 Implementation of a standard MSM . . . 7
A.8 Sensitivity analysis . . . 7

B System-specific methods **7**
B.1 Two spheres restrained by a multiscale spring potential . . . 7
B.1.1 System settings . . . 7
B.1.2 mMSM parameters . . . 8
B.2 Alanine dipeptide discretized based on the ϕ, ψ angles . . . 8
B.2.1 System settings . . . 8
B.2.2 mMSM parameters . . . 8
B.3 Alanine dipeptide discretized based on all seven torsion angles . . . 9
B.3.1 System settings . . . 9
B.3.2 mMSM parameters . . . 9
B.4 Villin headpiece (HP35) . . . 9
B.4.1 System settings . . . 9
B.4.2 mMSM parameters . . . 9
B.4.3 Analysis notes . . . 10
B.5 Evaluation of correctness of thermodynamic and kinetic properties . . . 10

C Supplementary figures **11**
Supplementary Figure S1 . . . 11
Supplementary Figure S2 . . . 12
Supplementary Figure S3 . . . 13
Supplementary Figure S4 . . . 14
Supplementary Figure S5 . . . 15
Supplementary Figure S6 . . . 16
Supplementary Figure S7 . . . 17
Supplementary Figure S8 . . . 18
Supplementary Figure S9 . . . 19
Supplementary Figure S10 . . . 20
Supplementary Figure S11 . . . 21
Supplementary Figure S12 . . . 22
Supplementary Figure S13 . . . 23
Supplementary Figure S14 . . . 24
Supplementary Table S1 . . . 25

Supplementary Table S2 26
Supplementary Table S3 27

A Supplementary methods

A.1 A standard Markov State Model (MSM)

A standard MSM represents a system's dynamics probabilistically by quantifying the conditional probabilities of transitioning between system states [1]. Formally, an MSM is specified by a set of system states $M = \{S_1, \dots, S_n\}$, representing a finite discretization of the configuration space; and an $n \times n$ transition matrix $T(\tau)$ or simply T, whose elements T_{ij} represent the probability of transitioning from S_i to S_j in time τ:

$$T_{ij} = Pr\left[S(t+\tau) = S_j \mid S(t) = S_i\right]$$

where $S(t)$ is the state of the system at time t. By design, this model implies that the transition probabilities depend only on the current state and not the preceding trajectory, a property also known as the Markov property. In this paper, we focus on the finite-state discrete-time setting. This setting was shown to provide useful descriptions for some key biomolecular processes [2, 3, 4], though theory for the infinite and continuous cases has been suggested [5].

Constructing an MSM. MSMs can be constructed in a number of ways. In a basic workflow, the system is first simulated for a certain number of steps, yielding one or more trajectories. A trajectory is specified by a sequence of consecutive configurations separated by some predetermined lag time. These configurations are mapped to a discrete set of system states, typically corresponding to compact volumes in the configuration space. Next, conditional transition probabilities are estimated from the discrete state trajectories. This process is often integrated with adaptive sampling, where the states of an intermediate MSM are used to select starting configurations for new simulation trajectories, which are in turn used to update the MSM, after which the process is repeated [6, 1].

A.2 mMSM-explore

The input to mMSM-explore is a starting configuration x_{init}. We assume that mMSM-explore can simulate the system using an external simulator (Simulator() in pseudocode notation). Given a system configuration x, the external simulator outputs k trajectories $\{\gamma_i\}_{i=1}^{k}$, where each trajectory γ_i is specified as a sequence of m configurations $(x_1, \dots, x_m)$, sampled at fixed time intervals τ_0 (the basic lag time), and starting from $x_1 = x$. mMSM-explore is also provided with a discretization scheme for generating microstates, or formally, a function that maps a continuous configuration x to a discrete microstate $\texttt{microstate}(x)$ (Section A.4). The mMSM is initialized using x_{init}, by setting the base set M^0 to $\{S_1^0\}$, where $S_1^0 = \texttt{microstate}(x_{\text{init}})$, and setting the set M^1 to the single trivial root macrostate S_1^1, where $S_1^1 = \{S_1^0\}$. Subsequently, multiple trajectories of a certain time duration are initiated from that initial state using the external simulator. The generated trajectories are used to update the mMSM (Supplementary Algorithm S1), which involves two aspects: updating the transition model (Section A.2.1), and refining the hierarchical partition of states (Section A.2.2). Refining the partition involves recognizing metastable states, through the detection of sets we refer to as temporal communities, and amending the hierarchy according to these sets. Finally, we use a sampling procedure we term multiscale adaptive sampling, which utilizes the existing mMSM structure to strategically select initial configurations for subsequent simulations.

A.2.1 Estimation of transition model

Transition rates between microstates are estimated using a Maximum Likelihood Estimator: given a discrete trajectory $(s(1), \dots, s(m))$, which is a trajectory of microstates from the set $\left\{S_1^0, S_2^0, \dots, S_{n_0}^0\right\}$, transition events are aggregated in a count matrix, which is then row-normalized to yield a stochastic transition matrix:

$$T^0_{k,l} = \frac{\sum_{i=1}^{m-1} \mathbb{1}\left[s(i+1) = S^0_l \ , \ s(i) = S^0_k\right]}{\sum_d \sum_{i=1}^{m-1} \mathbb{1}\left[s(i+1) = S^0_d \ , \ s(i) = S^0_k\right]} \tag{S1}$$

Transition models for subsequent levels $\left(T^1, \ldots, T^H\right)$ are updated recursively, by order of increasing height:

$$T^h_{k,l} = \frac{\sum_{s \in S^h_k} \pi^{h-1}(s) \sum_{s' \in S^h_l} T^{h-1}_{s,s'}}{\sum_{s \in S^h_k} \pi^{h-1}(s)} \tag{S2}$$

where π^h is the stationary distribution of level h, which exists by assumption of ergodicity. Ideally, the stationary distribution is computed using the entire matrix T^{h-1}. However, to improve computational efficiency and enable localized updates of each macrostate, we instead use the *local* stationary distribution, which is derived from a reduced matrix involving the macrostate's children and their immediate neighbors. Transitions from these neighbors to states outside the macrostate's children are treated as self-transitions, ensuring that the reduced matrix remains a valid stochastic matrix. These steps are implemented in the function UPDATETRANSITIONMATRIX(). In our implementation, this function takes a macrostate S^h_u as input, and updates the transition probabilities to its neighbors in M^h. That is, it updates a single row in T^h corresponding to S^h_u. Additionally, this procedure updates a reduced transition matrix over the children of S^h_u, which is used when partitioning to temporal communities (Section A.2.2).

A.2.2 Partitioning to temporal communities

The partitions represented by the macrostate sets $\left(M^1, \ldots, M^{H-1}\right)$ arise from identifying metastable states at the corresponding timescales $(\tau_1, \ldots, \tau_{H-1})$. In MSMs, the transition matrix T can be interpreted as a weighted directed graph: the nodes are the states of the system and the edge weights correspond to transition probabilities. We define *temporal communities* as subsets of states where internal transitions occur much faster than transitions to states outside the subset. These sets align with the notion of metastable states. It follows that partitioning the graph induced by $(T)^\kappa$ yields temporal communities that are metastable with respect to a longer lag time $\kappa \cdot \tau$, where τ is the lag time associated with T.

We employ a modularity-based graph clustering method to partition the MSM graph into temporal communities. The modularity score of a graph with respect to some node partition is a measure of the partition's quality in dividing the vertices into well-separated clusters; high modularity implies densely connected communities, with relatively sparse intercluster connectivity. While optimizing this measure is an NP-hard problem, multiple algorithms [7, 8, 9] have proven successful in finding high modularity partitions effectively. This score can be defined for various types of graphs; in our setting, we use a formulation suited for directed graphs, where edge weights correspond to transition probabilities. The modularity score of partitioning M^h to M^{h+1} is given by:

$$Q(M^{h+1}) = \sum_{S^h_i, S^h_j \in M^h} \left(T^h_{i,j} - \frac{\sum_k T^h_{k,j}}{|M^h|}\right) \delta(S^h_i, S^h_j) \tag{S3}$$

where $\delta(S^h_i, S^h_j) = 1$ if S^h_i and S^h_j are children of the same macrostate in M^{h+1}, and 0 otherwise. Since Q is a sum over all node pairs, modifying the partition locally does not require recomputing the modularity for the entire graph. Instead, the modularity difference can be efficiently determined by considering only the affected nodes.

During the model update step, each level's partition is adjusted so that it reflects temporal communities with high modularity. Formally, the states of M^{h+1}, which are subsets of the states in M^h, should be the temporal communities of the graph induced by $\left(T^h\right)^\kappa$, where $\kappa = \frac{\tau_{h+1}}{\tau_h}$. Since we are continuously updating the model with new simulation data, our understanding of the system's dynamics may evolve over time. Consequently, these changes should be reflected by the state partitions. The communities are maintained by continuously splitting or merging communities. After updating

the transition model from new simulation data, we examine all the macrostates containing each of the microstates encountered in the data, by order of increasing height. For each macrostate S_u^h, we choose one of three actions according to their gain in modularity. The first is splitting, which involves partitioning the children of S_u^h into temporal communities. The target graph is the one induced by restricting the transition matrix to the children of S_u^h and raising it to the appropriate power. The resulting communities will form new macrostates in place of S_u^h, with members of the communities as their children. The second possible action is merging S_u^h with one of its neighbors. This action reassigns S_u^h's children to the chosen neighbor. The third action will be to retain the current partition, if the other actions only decrease the overall modularity score.

A.2.3 Implementation details

Ideally, when updating the model with new simulation data, we should evaluate all relevant macrostates to determine whether they should be split or merged to maintain high modularity. However, to improve computational efficiency and ensure partition stability (e.g. preventing macrostates from being repeatedly merged and split), our implementation of the update procedure (Supplementary Algorithm S1) includes several modifications.

Specifically, when iterating the priority queue of macrostates to be updated (Supplementary Algorithm S1, lines 15–16), the priority criterion is a tuple of $(\textit{height}, \textit{action})$ (rather than just height), where *action* is 1 if the macrostate's transition probabilities were updated using UpdateTransitionMatrix(), and 0 otherwise. This ensures that relevant transition rates for an entire level were updated before proceeding to partition refinement. Once a macrostate is updated using UpdateTransitionMatrix(), it is reinserted to the queue with priority $(\textit{height}, 1)$. Additionally, each macrostate is considered for merging or splitting at most once during each update phase (that is, a single call to the UpdateMultiscaleMSM() procedure), and specific conditions are considered before attempting to split or merge it:

1. If macrostate S is the parent of another macrostate that was split or merged in the current phase, attempt to *split* S.
2. If not, examine the local MSM graph of S over its child nodes. If the graph's diameter exceeds a predefined threshold, attempt to *split* S.
3. If neither condition is met, attempt to *merge* S with one of its neighboring macrostates.

Splitting a macrostate involves applying the Leiden algorithm to its local MSM graph. We also added the option to randomly attempt splitting a non-root macrostate regardless of these heuristic conditions, according to some probability hyperparameter.

The lag times $\tau_1, \ldots, \tau_H$ associated with each MSM level dictate the timescales for which metastable states are recognized, with τ_0 being the basic lag time for transitions at the finest-grained (e.g. atomic) level. While these lag times can be chosen in advance through prior knowledge of the system, in our implementation they are chosen adaptively as simulation progresses, using a uniform temporal coarse-graining factor κ (here, $\kappa = 2$ by default). We set $\tau_1 = \kappa \cdot \tau_0$ for the initial level, M^1. If the root macrostate is split during the algorithm, the lag time of the new level is set to $\tau_2 = \kappa^2 \cdot \tau_0$ (with a new root macrostate), and so on. We stop introducing new levels and timescales once the highest level corresponds to the longest system processes. As shown in previous works, the implied timescales of a transition matrix, derived from its eigenvalues, are closely related to the stochastic processes it describes, particularly those governing the crossing of energy barriers [1]. Thus, we only split the root macrostate if its local transition matrix exhibits more than one magnitude of timescales. This step guarantees that the highest level of the hierarchy reflects the longest process in the system, preventing the introduction of unnecessary additional levels.

A.3 Multiscale adaptive sampling

Below, we outline the sampling distribution used in our implementation, which directs simulation toward underexplored regions.

Supplementary Algorithm S1 UPDATEMULTISCALEMSM

Input:

`mmsm`, an mMSM data structure

Γ, a set of k simulation trajectories $\{(x_{j,1}, \ldots, x_{j,m})\}_{j=1}^{k}$

$C \leftarrow$ `mmsm.count_matrix`
$U \leftarrow \emptyset$
for $j \leftarrow 1$ to k **do**
    for $i \leftarrow 1$ to $m-1$ **do**
        $S_{\text{from}} \leftarrow$ `microstate`$(x_{j,i})$
        $S_{\text{to}} \leftarrow$ `microstate`$(x_{j,i+1})$
        if $S_{\text{to}} \notin$ `mmsm.`M^0 **then**
            `mmsm.`$M^0 \leftarrow$ `mmsm.`$M^0 \cup \{S_{\text{to}}\}$
            S_{to}`.parent` $\leftarrow S_{\text{from}}$`.parent`
        end if
        $C[S_{\text{from}}, S_{\text{to}}] \mathrel{+}= 1$
        $U \leftarrow U \cup S_{\text{from}}$`.parent`
    end for
end for
while $U \neq \emptyset$ **do**
    $S_u^h \leftarrow$ macrostate from U with minimal height
    $U \leftarrow U \setminus \{S_u^h\}$
    $U \leftarrow U \cup S_u^h$`.parent`
    UPDATETRANSITIONMATRIX(S_u^h)
    if `split`(S_u^h) **then**
        $\{A_1, \ldots, A_p\} \leftarrow$ PARTITION$(S_u^h$`.children`$)$
        for $i \leftarrow 1$ to p **do**
            `mmsm.`$M^h \leftarrow$ `mmsm.`$M^h \cup \{S_{u_i}^h\}$
            $S_{u_i}^h$`.parent` $\leftarrow S_u^h$`.parent`
            $S_{u_i}^h$`.children` $\leftarrow A_i$
        end for
        $U \leftarrow U \cup \{S_{u_1}^h, \ldots, S_{u_p}^h\} \cup S_u^h$`.neighbors`
        `mmsm.`$M^h \leftarrow$ `mmsm.`$M^h \setminus \{S_u^h\}$
    else
        $S_{\text{merge}}^h \leftarrow$ MERGE(S_u^h)
        if $S_{\text{merge}}^h \neq S_u^h$ **then**
            S_{merge}^h`.children` $\leftarrow S_{\text{merge}}^h$`.children` $\cup\, S_u^h$`.children`
            $U \leftarrow U \cup S_u^h$`.neighbors` $\cup\, S_{\text{merge}}^h$`.children`
            `mmsm.`$M^h \leftarrow$ `mmsm.`$M^h \setminus \{S_u^h\}$
        end if
    end if
end while

Given a macrostate S_{cur} and its set of children $D(S_{\text{cur}}) = \{S_1, \ldots, S_n\}$, the probability of sampling a child S_i is:

$$P_{\text{adap}}(S_i) = \delta \pi_{S_{\text{cur}}}(S_i) + (1-\delta)\frac{\exp(-N(S_i))}{Z} \tag{S4}$$

where $\pi_{S_{\text{cur}}}(S_i)$ is the local stationary probability of S_i, $N(S_i)$ the total number of visits to microstates in the subtree rooted at S_i, δ a hyperparameter and $Z = \sum_{S_i \in D(S_{\text{cur}})} \exp(-N(S_i))$.

We designed this sampling rule to focus exploration on unexplored regions while avoiding highly improbable singular states. By sampling from a coarse-grained representation of the energy landscape, we mitigate the influence of such extreme states (Supplementary Figure S1). This approach can also improve computational efficiency. For instance, directly sampling a microstate using Equation S4 (as in a standard MSM) requires computing the stationary distribution of the full transition matrix. In contrast, the multiscale approach only requires computing stationary distributions within several small subsets of states.

A.4 Discretization scheme

We employ the K-centers algorithm [10] to discretize the configuration space into microstates. Given a set of points and a cutoff distance, the algorithm selects cluster centers such that every point lies within the cutoff distance of at least one center. These clusters define the microstates of the mMSM. Our implementation extends the algorithm to incorporate additional input points (obtained from further simulations), which are either assigned to existing clusters or used to form new clusters as needed. The function `microstate`(x) denotes the microstate to which configuration x is assigned or, if necessary, a newly created microstate accommodating x.

In adaptive sampling, when selecting initial configurations for simulations, we extract a representative configuration from the chosen microstate. Our discretization scheme uses reservoir sampling to maintain a set of representatives for each microstate.

A.5 Discretization error

Discretizing the continuous configuration space, often combined with dimensionality reduction, can introduce errors when quantifying dynamic properties [11, 12]. These errors arise when the partitioned states do not strictly satisfy the Markov property. In particular, initiating short trajectories from non-equilibrated configurations can bias the estimated transition rates and stationary probabilities. Although partitions can be rigorously Markovian under very specific conditions [13], these are rarely met in practice. Consequently, careful tuning of the lag time and state volume remains critical for minimizing the discretization error and recovering approximate Markovian behavior. A common test to validate lag time choices is an *implied-timescales convergence plot*; these were performed for the analysis of the two-spheres system and the alanine dipeptide (discretized on ϕ, ψ) system (Supplementary Figure S9).

A.6 Additional procedures

This section describes the sub-procedures used in the main algorithm.

- NewMultiscaleMSM(x): Initializes a Multiscale MSM structure. It takes a system configuration x as input, projects it onto the discrete space using the discretization method, and forms the first microstate. The result is a two-level structure consisting of a single microstate and a trivial root macrostate.

- Simulator(x, k, m): Given a configuration x, this subprocedure runs the system's simulator for $m-1$ steps, generating a trajectory of m configurations. It repeats this process k times, returning $\{\gamma_i\}_{i=1}^{k}$, a set of k trajectories.

- UpdateTransitionMatrix(S). Updates the outgoing transition probabilities from macrostate S, as described in Section A.2.1.

- `split`(S). Determines whether macrostate S should be split, based on the criteria described in Section A.2.2.
- Partition(S.children): Applies the Leiden graph clustering algorithm to the subgraph induced by the transition matrix over the children of S. The algorithm produces a partition $\{A_1, \ldots, A_p\}$, where each A_i is a subset of one or more child states. It also handles the case where the root macrostate is split (Section A.2.3) and a new root (with larger lag time) is introduced.
- Merge(S): Evaluates whether macrostate S should be merged with one of its neighboring macrostates. This is determined by calculating the modularity gain from merging S's children with those of its neighbor. The procedure returns the neighbor that yields the highest modularity gain or S itself if no merge results in an improvement.

A.7 Implementation of a standard MSM

For comparison purposes, we implemented a standard MSM by modifying our code to omit the hierarchical partitioning steps. Specifically, we restricted the algorithm to a single scale, where the MSM consists only of the microstates in M^0 with their transition matrix T^0 along with a trivial macrostate in M^1. As a result, the multiscale adaptive sampling procedure selects microstates directly, similar to conventional adaptive sampling methods.

A.8 Sensitivity analysis

We assessed the sensitivity of our results for the two-spheres system with respect to two key modeling parameters, while holding all other parameters fixed, repeating the full pipeline 10 times per setting: (i) the K-centers clustering cutoff radius that defines microstates in the discretization scheme (Supplementary Figure S10), and (ii) the graph-diameter threshold (in number of edges) that triggers a split of a macrostate (Supplementary Figure S11). For each setting, we report the mean number of macrostates at each level, the overall number of levels in the hierarchy per run, and the value of the d coordinate delimiting the two main basins. Across both parameter sweeps, the numbers of macrostates and hierarchy heights are mostly consistent, with increasing variance only at extreme values. The inferred delimiter remains tightly concentrated around $d = 39$ Å, consistent with the reference barrier.

While these results indicate robustness, parameter selection still requires care. The clustering radius should be paired with an appropriate lag time; mismatched choices can impair approximate Markovianity and destabilize the inferred partitions (the lag time was held fixed in this analysis). Supplementary Figure S10 shows that small cutoff radii produce more macrostates at the lowest levels of the hierarchy (immediately above the microstate discretization). At higher levels, the number of macrostates converges to similar values across radii, likely because those levels align with the deepest free-energy basins (see Figure 3, main text). In addition, stochastic variability in trajectory generation (simulation noise) can influence the learned partitions; poor parameter choices may amplify this variability and yield unstable macrostates. In practice, one can mitigate these effects by combining standard validation (e.g., implied-timescale and Chapman–Kolmogorov checks) with replication across independent simulations or random seeds, and by seeking consensus partitions over a modest range of parameter settings.

B System-specific methods

B.1 Two spheres restrained by a multiscale spring potential

B.1.1 System settings

The system was designed and simulated using the Integrative Modeling Platform (IMP) [14]. The potential energy function, which depends on the mutual distance between the spheres (d), is defined as:

$$f(d) = 45 \cdot 10^{-13} \cdot \mathcal{B}(d, 22.75, 0.06) \cdot \mathcal{B}(d, 27, 0.05) \cdot \mathcal{B}(d, 34, 0.04) \cdot \mathcal{B}(d, 44, 0) \cdot \mathcal{B}(d, 51, 0) \cdot \mathcal{B}(d, 55.25, 0) + 1.2 \sin(8d) + 2$$

where

$$\mathcal{B}(d, a, b) = (d - a)^2 - b$$

Naive simulations were performed by generating a single long trajectory of system configurations, using a basic lag time of $\tau_0 = 0.03$ ps. The configurations were binned, according to the mutual distances (d), into intervals of approximately 0.1 Å, covering the range from $d = 21.8$ Å to $d = 56.2$ Å.

B.1.2 mMSM parameters

We used our implementation of the K-centers algorithm to discretize the d-space, with a cluster diameter of approximately 0.1 Å. In the mMSM-explore generation loop, one ($k = 1$) trajectory of $m = 10^4$ steps, using a basic lag time of $\tau_0 = 0.03$ ps, was simulated from each initial configuration, with $n_{\text{init}} = 5$ initial configurations sampled per loop (except in the first loop). The multiscale adaptive sampling distribution was weighted as follows:

$$P_{\text{adap}}(S_i) = 0.3 \cdot \pi_{S_{\text{cur}}}(S_i) + 0.7 \cdot \frac{\exp(-N(S_i))}{Z}$$

where P_{adap} is used to sample a child of S_{cur}, $\pi_{S_{\text{cur}}}(S_i)$ is the probability of S_i in the local stationary distribution over the children of S_{cur}, $N(S_i)$ is the total number of visits to microstates in the subtree rooted at S_i, and

$$Z = \sum_{S_i \in D(S_{\text{cur}})} \exp(-N(S_i))$$

is the normalization factor.

The diameter threshold for splitting macrostates (Section A.2.2) was set to 4 edges. The probability of attempting a random split of a non-root macrostate (Section A.2.3) was set to 0.05.

B.2 Alanine dipeptide discretized based on the ϕ, ψ angles

B.2.1 System settings

The system consists of a single alanine amino acid, capped with an acetyl group at the N-terminus and an N-methylamide group at the C-terminus. We used the AMBER software package [15] and the AMBER ff14SB force field to define the system topology and forces. Simulations were performed using the OpenMM package [16].

Naive simulations were conducted by generating a single long trajectory of system configurations, using a basic lag time of $\tau_0 = 0.04$ ps. The dihedral angles (ϕ, ψ) from these configurations were binned into a grid covering the range of $-180°$ to $180°$ for each angle, with approximately $3° \times 3°$ sized bins.

B.2.2 mMSM parameters

We used our implementation of the K-centers algorithm to discretize the (ϕ, ψ) space, using a cluster diameter of approximately 6°. In the mMSM-explore generation loop, one ($k = 1$) trajectory of $m = 10^3$ steps, with a basic lag time of $\tau_0 = 0.04$ ps, was simulated from each initial configuration, and $n_{\text{init}} = 4$ initial configurations sampled per loop (except in the first loop). The multiscale adaptive sampling distribution was weighted as follows:

$$P_{\text{adap}}(S_i) = 0.4 \cdot \pi_{S_{\text{cur}}}(S_i) + 0.6 \cdot \frac{\exp(-N(S_i))}{Z}$$

where P_{adap} is used to sample a child of S_{cur}, $\pi_{S_{\text{cur}}}(S_i)$ is the probability of S_i in the local stationary distribution over the children of S_{cur}, $N(S_i)$ is the total number of visits to microstates in the subtree rooted at S_i, and

$$Z = \sum_{S_i \in D(S_{\text{cur}})} \exp\left(-N(S_i)\right)$$

is the normalization factor.

The diameter threshold for splitting macrostates (Section A.2.2) was set to 8 edges.

B.3 Alanine dipeptide discretized based on all seven torsion angles

B.3.1 System settings

The simulation settings and setup were identical to those in Section B.2.

B.3.2 mMSM parameters

We used the K-centers algorithm to discretize the 7-dimensional dihedral angles space, using a cluster diameter of approximately 120°. In the mMSM-explore generation loop, one ($k = 1$) trajectory of $m = 10^3$ steps, with a basic lag time of $\tau_0 = 0.1$ ps, was simulated from each initial configuration, with $n_{\text{init}} = 14$ initial configurations sampled per loop (except in the first loop).

The sampling distribution for the multiscale adaptive sampling procedure was equilibrium only, that is:

$$P_{\text{adap}}(S_i) = \pi_{S_{\text{cur}}}(S_i)$$

where P_{adap} is used to sample a child of S_{cur}, and $\pi_{S_{\text{cur}}}(S_i)$ is the probability of S_i in the local stationary distribution over the children of S_{cur}.

The diameter threshold for splitting macrostates (Section A.2.2) was set to 8 edges.

B.4 Villin headpiece (HP35)

B.4.1 System settings

We analyzed the 300 µs HP35 trajectory by Piana *et al.* [17], simulated in explicit solvent with the Amber ff99SB*-ILDN force field [18, 19, 20] and TIP3P water [21] at 360 K, with frames saved every 0.2 ns. Nagel *et al.* [22] converted each frame to a 42-dimensional vector of *native contact* distances. Native contacts are residue pairs at least 4 residues apart, whose heavy atoms are within 4.5 Å in at least 30% of the trajectory. In each converted frame, a contact distance records the minimal heavy-atom distance between the residues[1]. Further pre-processing includes Gaussian low-pass filtering ($\sigma = 10$ ns), standardization, and dimensionality reduction using PCA to 10 components. The processed trajectory is streamed in consecutive chunks into mMSM-explore to construct the hierarchical mMSM.

B.4.2 mMSM parameters

We used our implementation of the K-centers algorithm to discretize the 10-dimensional space, using a cluster diameter of 3.5. In the mMSM-explore generation loop, a single trajectory of $m = 10^5$ steps is given as input. Transition estimation was done with a lag time of 100 ns (500 steps of 0.2 ns) using a sliding-window across the trajectory. The diameter threshold for splitting macrostates (Section A.2.2) was set to 8 edges. Since we were using a precomputed trajectory, parameters associated with adaptive sampling procedures (number of initial configurations, sampling distributions) were not used.

[1] The minimal distance is computed over atom pairs whose distance is under 4.5 Å in at least 30% of the population.

B.4.3 Analysis notes

Calculation of Q For each 42-dimensional vector of native contact distances, Q is defined as the fraction of distances that are smaller than 4.5 Å. To determine the Q population for a particular macrostate, we inspect the microstates composing that macrostate. Each microstate retains a set of representative distance vectors, for which Q can be calculated; the union of all these sets determines the population of that particular macrostate.

B.5 Evaluation of correctness of thermodynamic and kinetic properties

Our use of the K-centers algorithm to discretize the configuration space, along with the inherent randomness in the temporal community detection procedures, introduces slight variations in the partitioning with each mMSM-explore run. These differences in macrostate partitions complicate the comparison of thermodynamic and kinetic properties across runs, as each run produces a distinct set of states. To ensure consistency in these comparisons, we manually standardized the partitioning when calculating stationary probabilities and transition rates. The sets of microstates for each run were manually grouped according to this standardized partition. For the two-spheres system, we used the partitioning of the six intermediate basins and two main basins depicted in Figure 3 of the main text. For alanine dipeptide, we used the partitioning of the five conformations shown in Figure 6C (M^3) of the main text.

C Supplementary figures

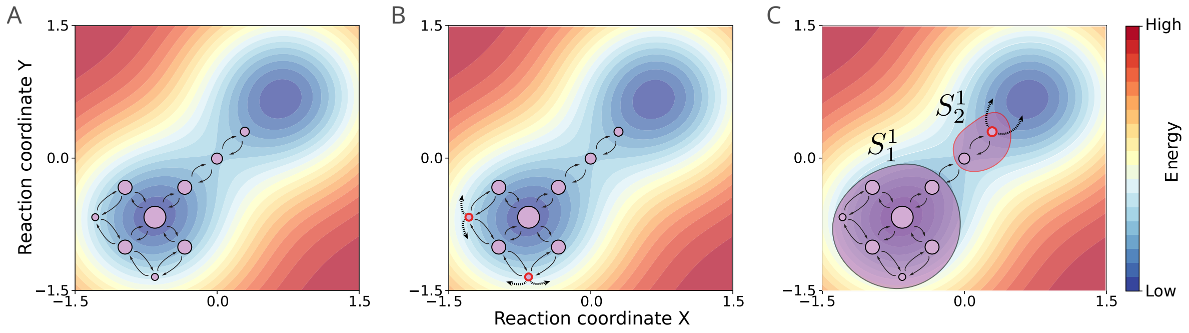

Supplementary Figure S1: Illustration of multiscale adaptive sampling. (A) A free energy landscape with two basins, generated from a mixture of two Gaussians. The pink vertices represent an MSM estimated from simulations that have explored the bottom-left basin and partially explored the top-right basin. Node sizes are proportional to their probability. To continue exploration, a starting point for further simulation should be selected, preferably a transition state or an undersampled state (i.e., a low-probability state). (B) Standard adaptive sampling. By targeting low-probability microstates, we are likely to select states near the boundary (red) of the bottom-left basin, which has already been extensively explored. Simulations initiated from these points are unlikely to reach new regions of the configuration space and will, in most cases, retract to the basin center. (C) Multiscale adaptive sampling. Instead of directly selecting a low-probability state, we first choose a macrostate at a higher level (purple). The left basin (S_1^1) has a higher probability because it has been mostly explored, whereas S_2^1 is relatively undersampled. As a result, we are more likely to select S_2^1 as the starting region and use its local MSM (pink) to initiate new simulations (red).

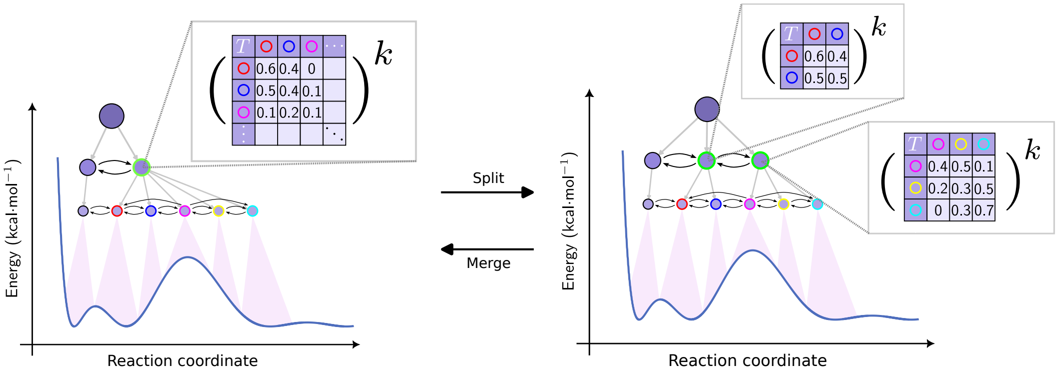

Supplementary Figure S2: The splitting and merging process of updated macrostates (light green), based on the transition dynamics among their children. The child states are either split into different temporal communities (left to right) or merged into a single one (right to left).

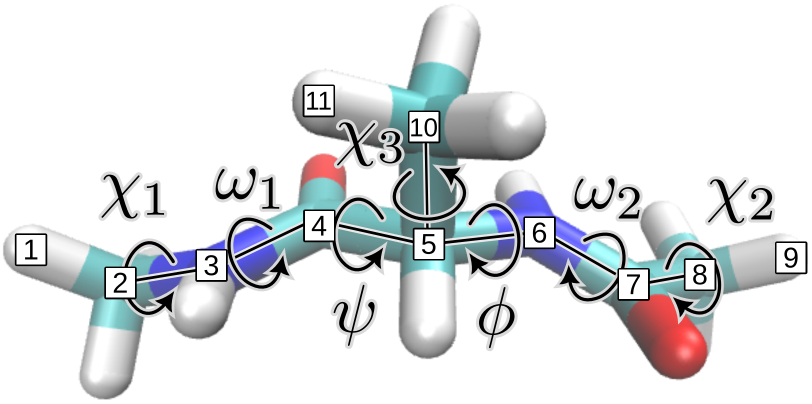

Supplementary Figure S3: Alanine dipeptide and the seven dihedral angles analyzed in this study. Backbone atoms are designated numerically. Exact atoms defining each dihedral angle are listed in Supplementary Table S1.

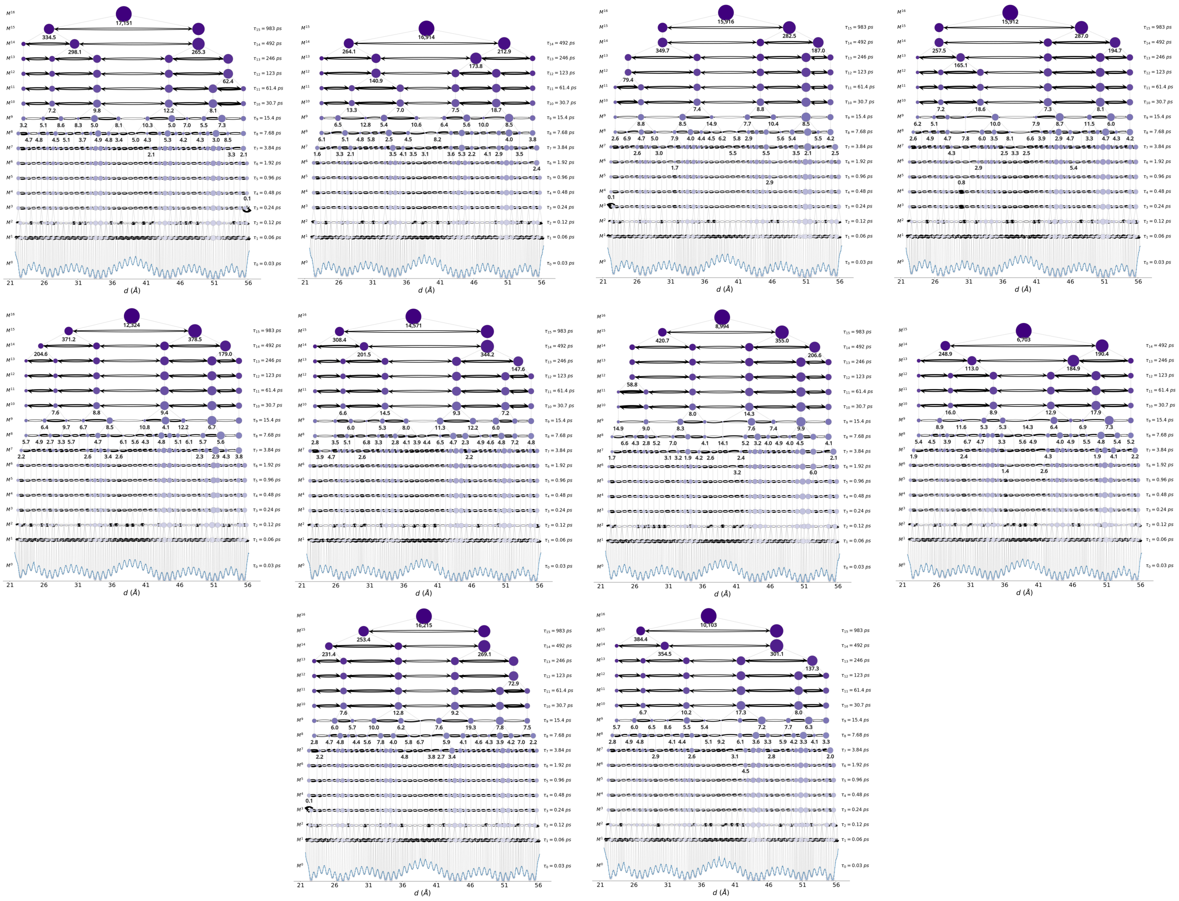

Supplementary Figure S4: The mMSM structures from each of the 10 independent mMSM-explore runs conducted for the two-spheres system, showing similar partitions to metastable states with minor differences.

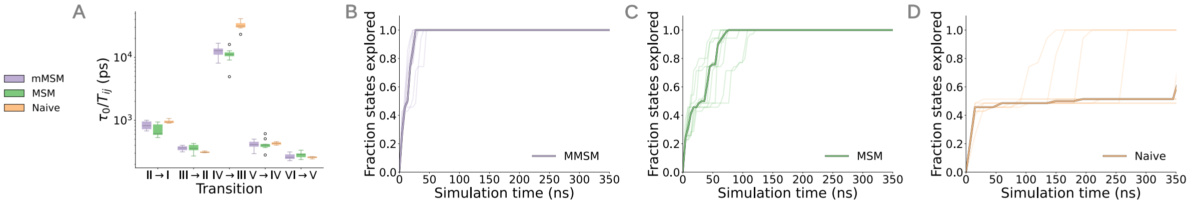

Supplementary Figure S5: Comparison of accuracy and exploration rates using mMSM (purple; 10 independent runs of 1,000 ns), MSM (green; 10 independent runs of 1,000 ns), and naive simulation (orange; 10 independent runs of 20,000 ns) for the two-spheres system (Section A.7, B.1). (A) Leftward one-step transition times between the six intermediate energy basins. Median values are indicated within each box, which spans the first to third quartiles, with whiskers extending 1.5 times the interquartile range. Naive estimates serve as the ground truth. (B–D) Exploration rate comparisons for each method. The full state space is defined as the interval between $d = 22\,\text{Å}$ and $d = 56\,\text{Å}$, and the fraction of visited states is plotted. For each method, the median value (opaque) and individual simulation values (transparent) are shown.

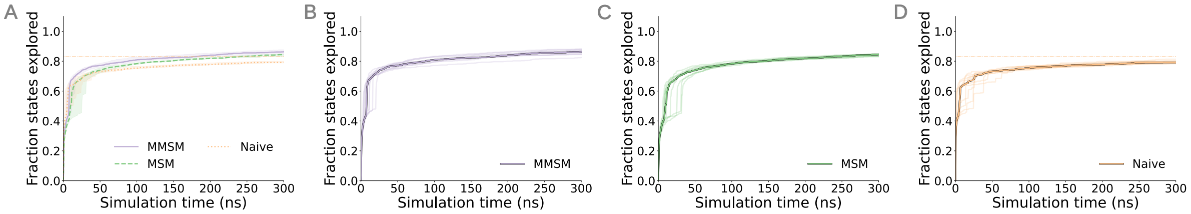

Supplementary Figure S6: Exploration rate comparisons for each method, in the alanine dipeptide system. We define 100% of the space as all the values between -180° and 180° for both ϕ and ψ, divided into a fine grid; the fraction of visited states is then plotted. (A) Median exploration rate with a 95% confidence interval. (B–D) For each method, the median value (opaque) and individual simulation values (transparent) are shown.

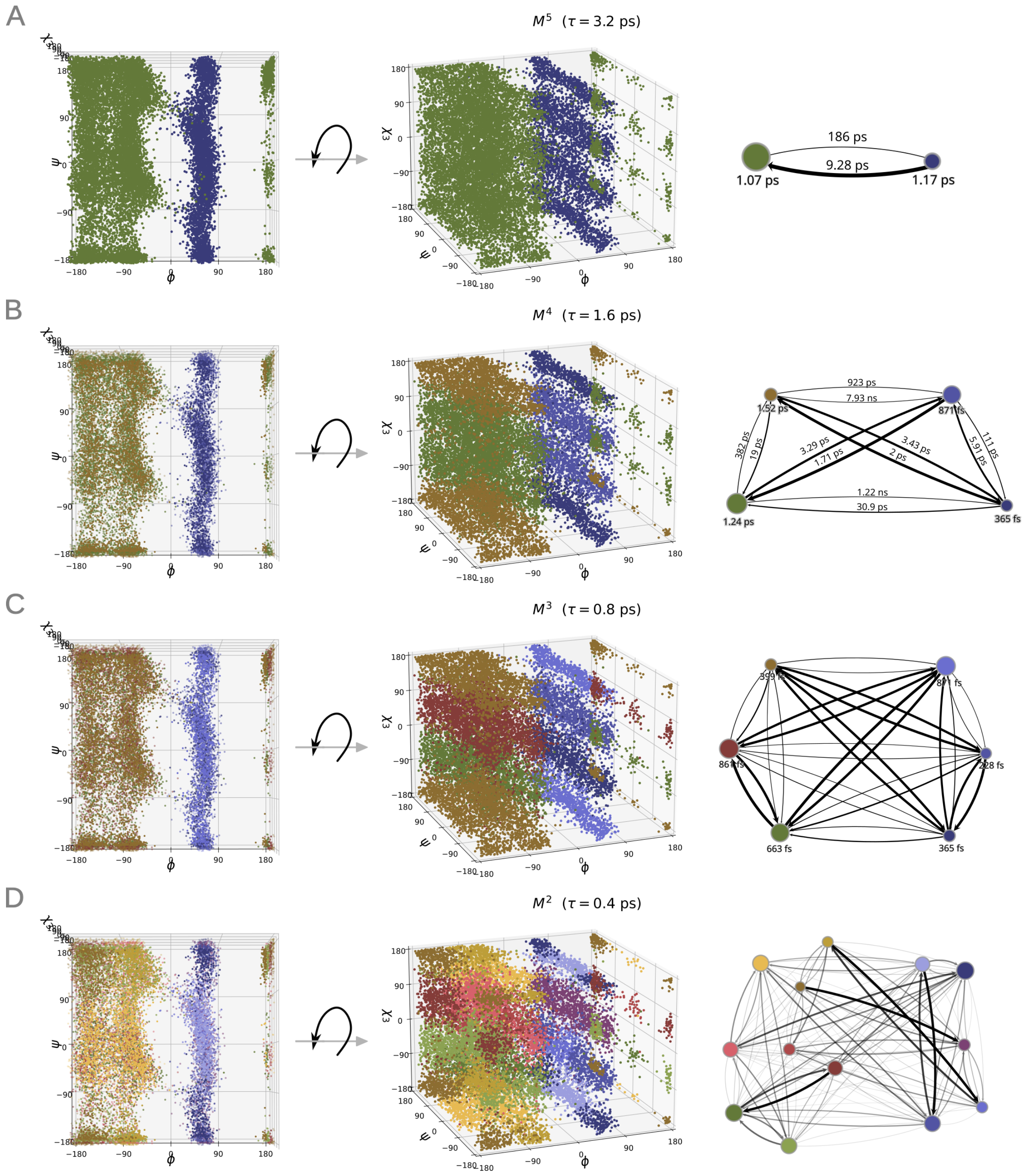

Supplementary Figure S7: Visualization of the representative mMSM, showing macrostate partitions at levels M^5 (A), M^4 (B), M^3 (C) and M^2 (D). In each figure, the left and center panels display two perspectives of the projection of microstates onto the ϕ, ψ, and χ_3 reaction coordinates. Colors indicate the assignment of microstates to macrostates at the respective level. The right panel depicts the corresponding macrostate graph, where nodes represent macrostates, colored as in the left panel. Node sizes are proportional to their stationary distribution probabilities. Edges indicate transition paths, with widths proportional to the transition rates over the basic lag time $\tau_0 = 0.1$ ps and their labels denote the one-step transition time. Self-transitions are omitted for clarity. Labels beneath the nodes represent local relaxation times. For readability, labels are omitted for (C) and (D). In (D), edge transparency is proportional to the transition rates, in addition to edge width.

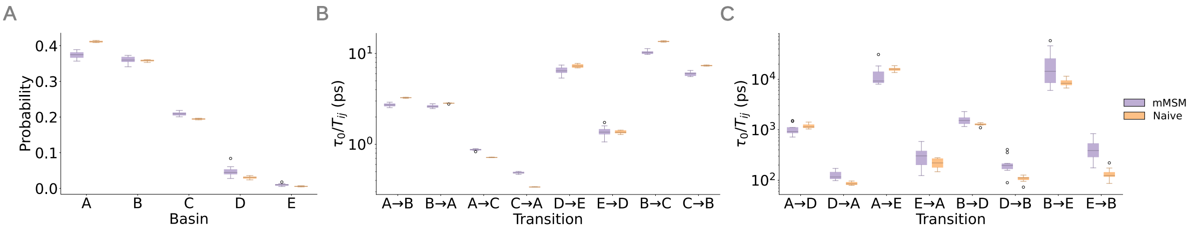

Supplementary Figure S8: Comparison of accuracy using mMSM (purple; 10 independent runs of 300 ns) and naive simulation (orange; 10 independent runs of $1,000$ ns) for the 7-dimensional mMSM-explore runs in the alanine dipeptide system, with respect to its five known states (designated in the main text). Median values are indicated within each box. The boxes span quantiles 1 to 3, with whiskers extending 1.5 times the interquartile range. The naive estimates are regarded as the ground truth values. (A) Stationary probabilities for each state. (B, C) One-step transition times for the possible transitions between the five states.

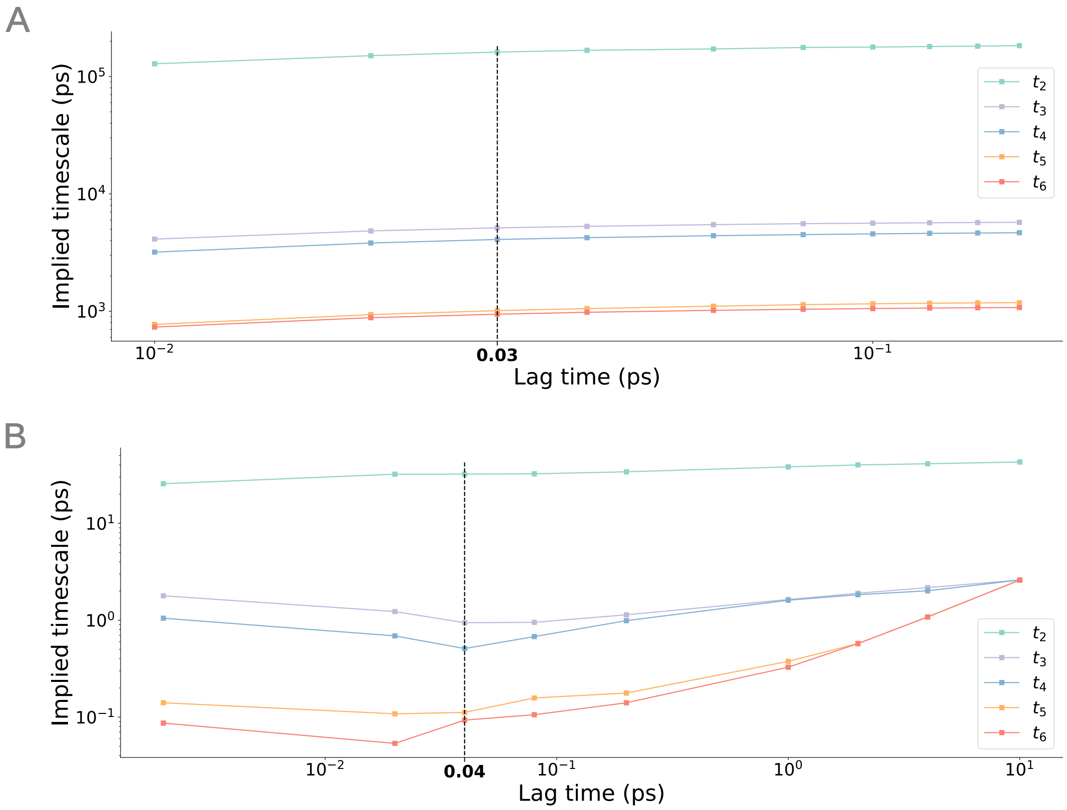

Supplementary Figure S9: Implied timescales convergence. Implied timescales t_i are plotted as a function of lag time τ for (A) the two-spheres system and (B) alanine dipeptide discretized based on ϕ, ψ. For a Markov state model estimated at lag time τ, the ith implied timescale is defined as $t_i(\tau) = -\tau / \ln \lambda_i(\tau)$, where $\lambda_i(\tau)$ is the ith eigenvalue of the corresponding transition matrix. The stationary mode t_1 is omitted; the curves show t_2 to t_6. Plateaus of $t_i(\tau)$ as τ increases indicate consistency of the discretization with Markovian dynamics at the chosen τ. Vertical dashed lines mark the lag time used in our analysis ($\tau_0 = 0.03$ ps in A; $\tau_0 = 0.04$ ps in B). For all τ values, transition matrices are estimated using the same fixed partition into microstates, obtained from a single run of our method in each of the two systems; the representatives appear in the main text (Figure 4 in A; Figure 6 in B). Estimates are based on independent, unbiased MD trajectories of 1 µs (two-spheres) and 2 µs (alanine dipeptide).

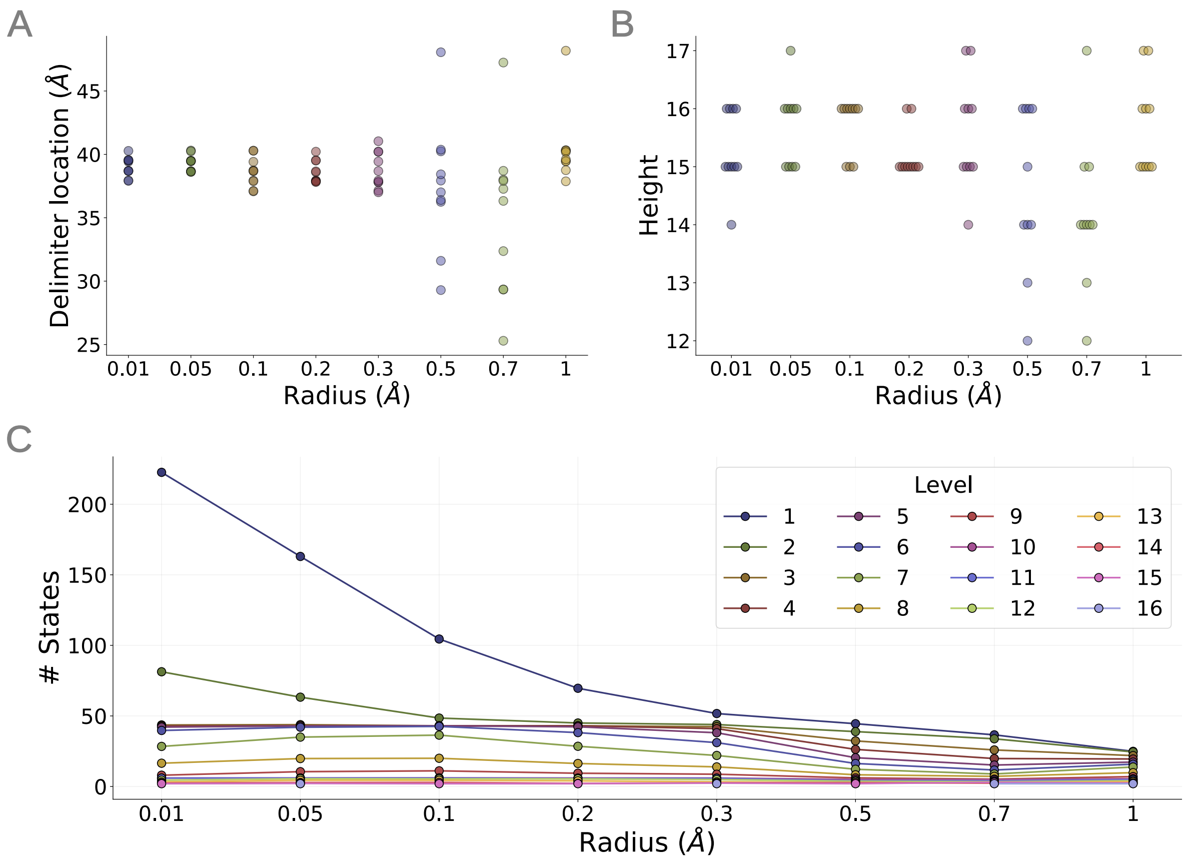

Supplementary Figure S10: Sensitivity to the clustering radius used for discretization, in the two-spheres system. (A) Location (in Å) of the delimiter in the distance coordinate separating the two main basins inferred at the top level, versus the cutoff radii. Each marker indicates an individual run. (B) Final mMSM height versus cutoff radii. Each marker indicates an individual run. (C) Mean number of macrostates at each mMSM level (legend) as a function of the cutoff radii, across 10 independent runs. Level 1 refers to M^1, the first level of macrostates. Discretization follows our K-centers scheme (Section A.4), and the two-spheres system is parameterized as described in Section B.1

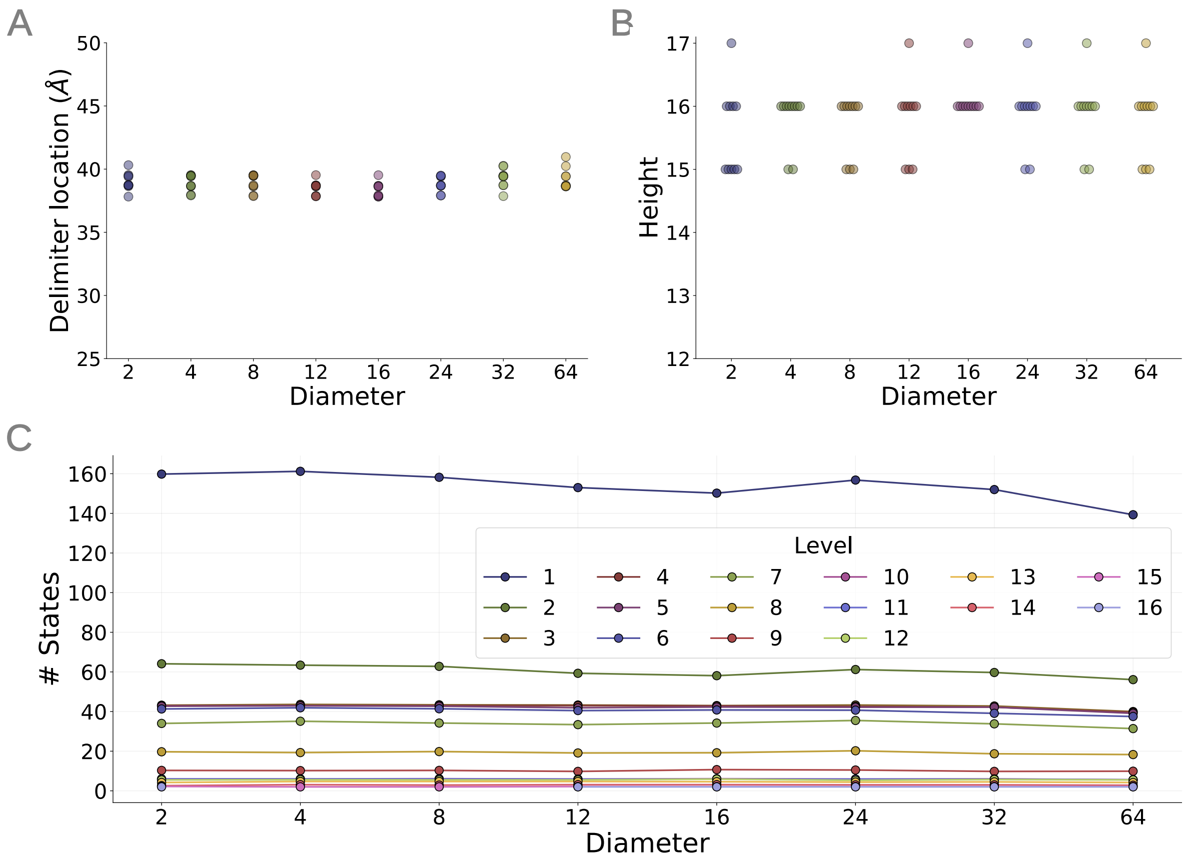

Supplementary Figure S11: Sensitivity to the graph diameter threshold used in the split heuristic (Section A.2.3), in the two-spheres system. (A) Location (in Å) of the delimiter in the distance coordinate separating the two main basins inferred at the top level, versus the threshold value. Each marker indicates an individual run. (B) Final mMSM height versus threshold value. Each marker indicates an individual run. (C) Mean number of macrostates at each mMSM level (legend) as a function of the diameter threshold, across 10 independent runs. Level 1 refers to M^1, the first level of macrostates. Discretization follows our K-centers scheme (Section A.4), and the two-spheres system is parameterized as described in Section B.1.

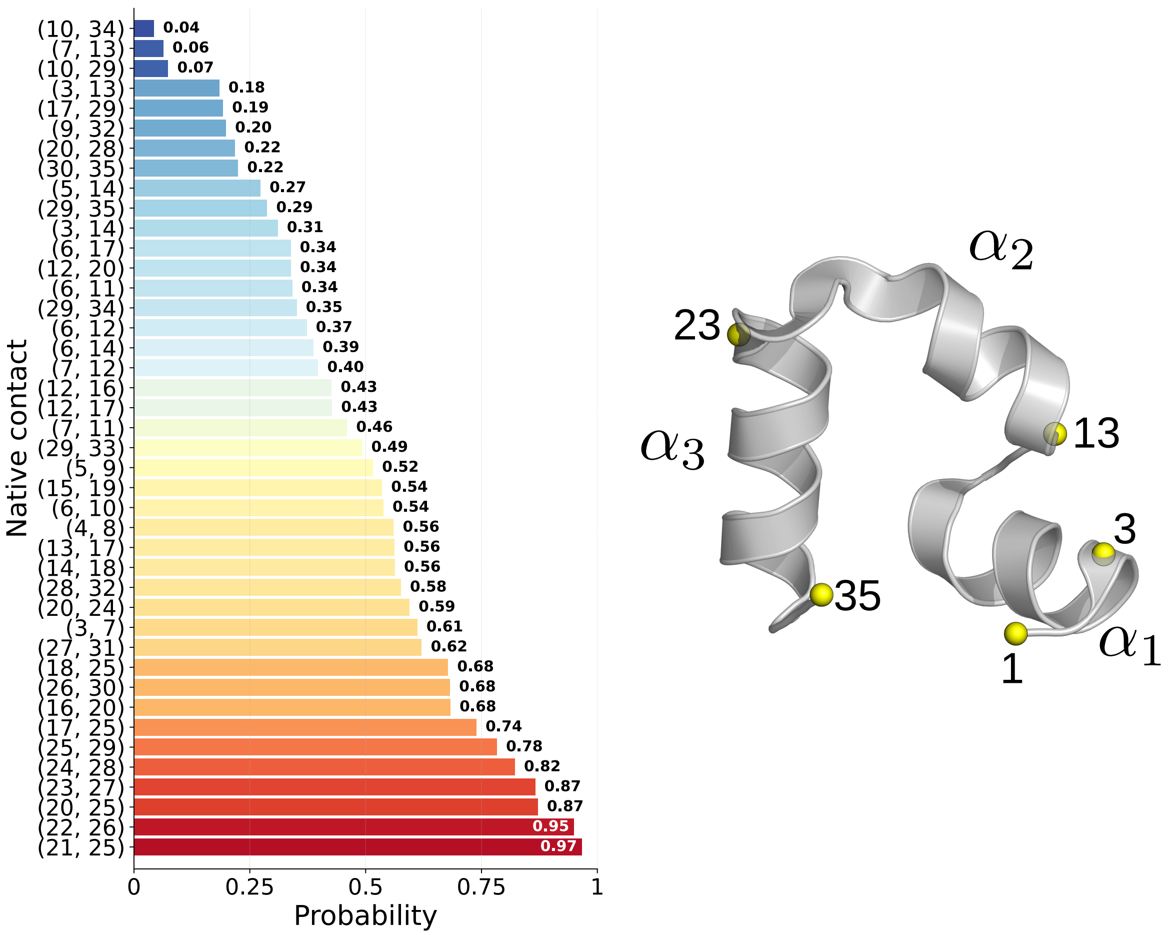

Supplementary Figure S12: Native-contact probabilities for the villin headpiece (HP35). **Left:** Formation probability for each of the 42 native contacts (i, j), where i and j are residue indices within the 35-residue sequence. The contacts are sorted by descending probability; bar color also encodes probability (blue = low, red = high). Probabilities were calculated from the mMSM's ensemble. **Right:** Cartoon representation of HP35 indicating helices α_1, α_2, and α_3; selected residues (1, 3, 13, 23, 35) are shown as spheres for orientation.

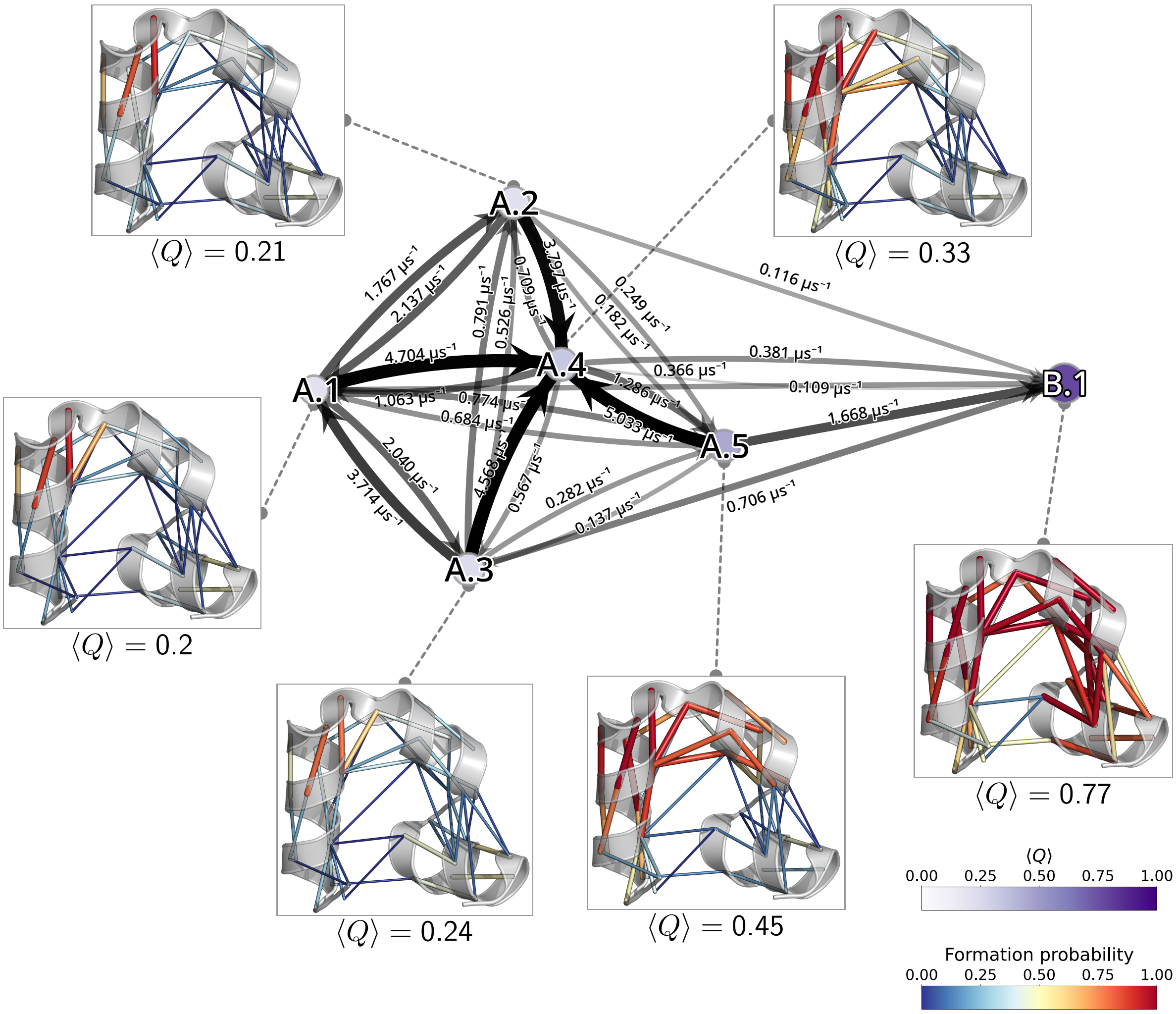

Supplementary Figure S13: Third level of the mMSM generated by mMSM-explore for the villin headpiece (HP35). Node size is proportional to stationary probability, node color encodes $\langle Q \rangle$. Edge width and opacity are proportional to transition probability. Edge labels indicate kinetic rates. For visibility, edges below a probability threshold (0.01) are omitted. Structure-based visualization of the 42 native contacts and their probabilities within each macrostate is shown; contact widths and colors are according to the formation probability within the macrostate.

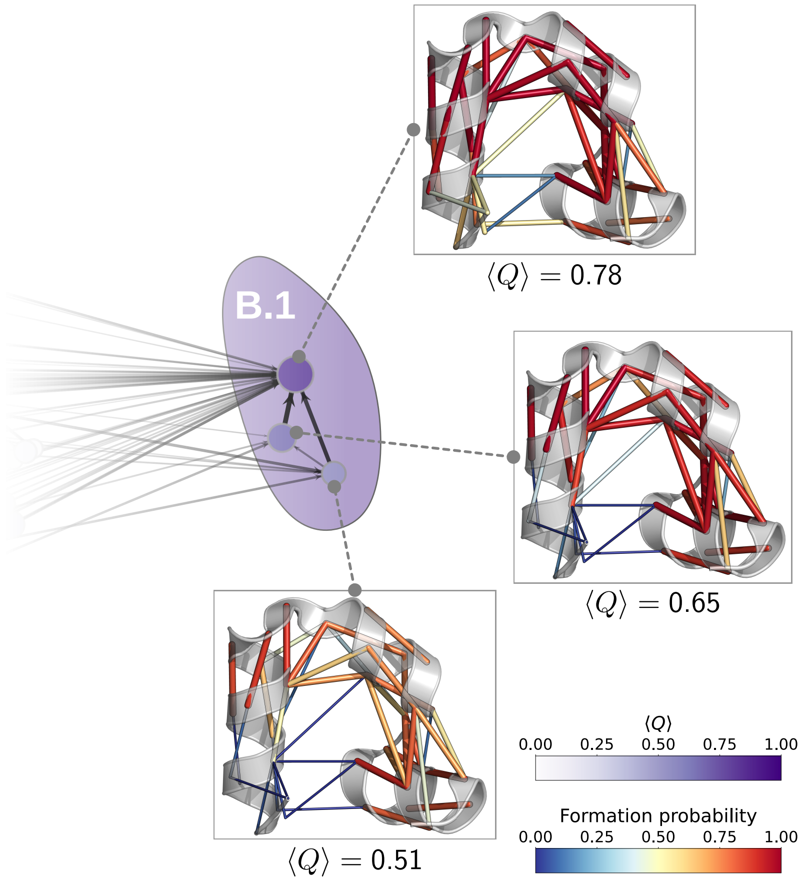

Supplementary Figure S14: Inner dynamics of the B.1 macrostate (from level 3), as resolved by the first level of the mMSM for the villin headpiece (HP35). Node size is proportional to stationary probability, node color encodes $\langle Q \rangle$. Edge width and opacity are proportional to transition probability. Edge labels indicate kinetic rates. For visibility, edges below a probability threshold (0.04) are omitted. Structure-based visualization of the 42 native contacts and their probabilities within each macrostate is shown; contact widths and colors are according to the formation probability within the macrostate.

Angle	Atoms
χ_1	1–2–3–4 (H–C–N–C)
ω_1	2–3–4–5 (C–C–N–C)
ψ	3–4–5–6 (N–C–C–N)
ϕ	4–5–6–7 (C–C–N–C)
ω_2	5–6–7–8 (C–N–C–C)
χ_2	6–7–8–9 (N–C–C–H)
χ_3	6–5–10–11 (N–C–C–H)

Supplementary Table S1: Dihedral angles analyzed in alanine dipeptide, along with the atom numbers and element types defining each angle. The numerical atom designations correspond to those shown in Supplementary Figure S3.

Method	# traj.[1]	Wall [h]	Core-h[2]	Peak RAM [GB][3]	Sim. [h]	Disc. [h]	Update [h]	Adapt. [h]
mMSM	14	0.354	4.34	0.42	0.341	0.0040	0.0072	$< 10^{-5}$
MSM	14	0.352	4.42	0.42	0.343	0.0039	0.0020	$< 10^{-5}$
Naive MD	1	9.05	56.9	1.81	8.98	0.0021	0.034	-

Supplementary Table S2: Runtime and memory benchmark for a 100 ns alanine dipeptide test-case. mMSM and standard MSM maintain 14 concurrent 1,000-step (with 40 fs lag time) trajectories, restarting them via adaptive sampling until an aggregate of 100 ns is collected. The naive protocol runs a single continuous trajectory, updating a count matrix every 2,000 steps (40 fs lag time). Wall-clock, CPU usage, and peak RAM were obtained with `/usr/bin/time -v`. All runs executed on an Intel Core Ultra 9 185H (22 logical cores).

[1] Maximum number of concurrent trajectories.

[2] Core-hours computed as (user time + system time), i.e. effective CPU time across all threads.

[3] Peak resident set size (RSS) reported by `/usr/bin/time -v`. The higher value for the naive run stems from storing the full count matrix in dense format, whereas a sparse representation is used in the mMSM and MSM implementations.

Method	Key assumption	Hierarchy depth	State discovery	Adaptive sampling[1]	Typical strength
mMSM (ours)	Distinct processes exist at multiple timescales; each timescale can be represented by an MSM level	Unlimited	Automatic, on-the-fly during simulation	Yes	Captures dynamics across multiple timescales with a hierarchical structure; allows unsupervised discovery of metastable states and adaptive steering across fast and slow processes.
Hi-MSM	Fast intra-basin vs. slow inter-basin dynamics; treat basins with local MSMs and connect them via a coarse network	Two levels	Local states learned within basins; inter-basin links built with COS/US; two-level structure fixed a priori.	No	Explicitly decouples fast intra-basin from slow inter-basin processes; enables modular, scalable modeling for systems with well-separated barriers.
VAMPnets	Slow modes can be spanned by a fixed set of fuzzy states learned by a neural network (VAMP objective)	Single level	Learned *post-simulation*; user fixes number of states	No/partially[2]	Bypasses traditional MSM pipeline (dimensionality reduction, discretization, validation); learns fuzzy state assignments end-to-end from raw data[3].
Coarse-grained MSM	Clear spectral gap allows lumping microstates without losing slow kinetics	Single level	Post-hoc aggregation of an MSM	Yes	Widely used and methodologically robust; output models are compact, interpretable, and supported by a mature validation framework.

Supplementary Table S3: Side-by-side qualitative comparison of four kinetic modeling frameworks: multiscale MSM (mMSM), Hierarchical MSM (Hi-MSM) [23], VAMPnets [24], and coarse-grained MSMs [1]. Columns summarise each method's key modeling assumption, hierarchy depth, state-discovery strategy, built-in suitability for adaptive sampling, and a distinctive practical strength.

[1] "Yes/No" indicates whether the method includes a built-in adaptive driver in the cited implementation; all methods are compatible with external adaptive-sampling frameworks.

[2] Adaptive-sampling variants such as *MaxEnt-VAMPnet*, [25] retrain the network between simulation rounds, although the workflow is still less straightforward than MSM-based adaptive sampling.

[3] A different but increasingly common use of VAMPnets is learning *reaction coordinates*, after which discretization and coarse-graining is done as in coarse-grained MSMs.

References

[1] Jan-Hendrik Prinz, Hao Wu, Marco Sarich, Bettina Keller, Martin Senne, Martin Held, John D Chodera, Christof Schütte, and Frank Noé. Markov models of molecular kinetics: Generation and validation. *The Journal of chemical physics*, 134(17):174105, 2011.

[2] Md Shafiqul Islam, Samuel L Junod, Si Zhang, Zakey Yusuf Buuh, Yifu Guan, Mi Zhao, Kishan H Kaneria, Parmila Kafley, Carson Cohen, Robert Maloney, et al. Unprotected peptide macrocyclization and stapling via a fluorine-thiol displacement reaction. *Nature Communications*, 13(1):350, 2022.

[3] Thomas J Lane, Gregory R Bowman, Kyle Beauchamp, Vincent A Voelz, and Vijay S Pande. Markov state model reveals folding and functional dynamics in ultra-long md trajectories. *Journal of the American Chemical Society*, 133(45):18413–18419, 2011.

[4] Neha Vithani, Tyson D Todd, Sukrit Singh, Tony Trent, Kendall J Blumer, and Gregory R Bowman. G protein activation occurs via a largely universal mechanism. *The Journal of Physical Chemistry B*, 128(15):3554–3562, 2024.

[5] William J Anderson. *Continuous-time Markov chains: An applications-oriented approach.* Springer Science & Business Media, 2012.

[6] Brooke E Husic and Vijay S Pande. Markov state models: From an art to a science. *Journal of the American Chemical Society*, 140(7):2386–2396, 2018.

[7] Vincent D Blondel, Jean-Loup Guillaume, Renaud Lambiotte, and Etienne Lefebvre. Fast unfolding of communities in large networks. *Journal of statistical mechanics: theory and experiment*, 2008(10):P10008, 2008.

[8] Vincent A Traag, Ludo Waltman, and Nees Jan Van Eck. From louvain to leiden: guaranteeing well-connected communities. *Scientific reports*, 9(1):5233, 2019.

[9] Aaron Clauset, Mark EJ Newman, and Cristopher Moore. Finding community structure in very large networks. *Physical review E*, 70(6):066111, 2004.

[10] Teofilo F Gonzalez. Clustering to minimize the maximum intercluster distance. *Theoretical computer science*, 38:293–306, 1985.

[11] Vijay S Pande, Kyle Beauchamp, and Gregory R Bowman. Everything you wanted to know about markov state models but were afraid to ask. *Methods*, 52(1):99–105, 2010.

[12] William C Swope, Jed W Pitera, and Frank Suits. Describing protein folding kinetics by molecular dynamics simulations. 1. theory. *The Journal of Physical Chemistry B*, 108(21):6571–6581, 2004.

[13] Jianjun Paul Tian and Dan Kannan. Lumpability and commutativity of markov processes. *Stochastic analysis and Applications*, 24(3):685–702, 2006.

[14] Daniel Russel, Keren Lasker, Ben Webb, Javier Velázquez-Muriel, Elina Tjioe, Dina Schneidman-Duhovny, Bret Peterson, and Andrej Sali. Putting the pieces together: integrative modeling platform software for structure determination of macromolecular assemblies. *PLoS biology*, 10(1):e1001244, 2012.

[15] David A Case, H Metin Aktulga, Kellon Belfon, Ido Ben-Shalom, Scott R Brozell, David S Cerutti, Thomas E Cheatham III, Vinícius Wilian D Cruzeiro, Tom A Darden, Robert E Duke, et al. *Amber 2021.* University of California, San Francisco, 2021.

[16] Peter Eastman, Jason Swails, John D Chodera, Robert T McGibbon, Yutong Zhao, Kyle A Beauchamp, Lee-Ping Wang, Andrew C Simmonett, Matthew P Harrigan, Chaya D Stern, et al. Openmm 7: Rapid development of high performance algorithms for molecular dynamics. *PLoS computational biology*, 13(7):e1005659, 2017.

[17] Stefano Piana, Kresten Lindorff-Larsen, and David E Shaw. Protein folding kinetics and thermodynamics from atomistic simulation. *Proceedings of the National Academy of Sciences*, 109(44):17845–17850, 2012.

[18] Viktor Hornak, Robert Abel, Asim Okur, Bentley Strockbine, Adrian Roitberg, and Carlos Simmerling. Comparison of multiple amber force fields and development of improved protein backbone parameters. *Proteins: Structure, Function, and Bioinformatics*, 65(3):712–725, 2006.

[19] Robert B Best and Gerhard Hummer. Optimized molecular dynamics force fields applied to the helix- coil transition of polypeptides. *The journal of physical chemistry B*, 113(26):9004–9015, 2009.

[20] Kresten Lindorff-Larsen, Stefano Piana, Kim Palmo, Paul Maragakis, John L Klepeis, Ron O Dror, and David E Shaw. Improved side-chain torsion potentials for the amber ff99sb protein force field. *Proteins: Structure, Function, and Bioinformatics*, 78(8):1950–1958, 2010.

[21] William L Jorgensen, Jayaraman Chandrasekhar, Jeffry D Madura, Roger W Impey, and Michael L Klein. Comparison of simple potential functions for simulating liquid water. *The Journal of chemical physics*, 79(2):926–935, 1983.

[22] Daniel Nagel, Sofia Sartore, and Gerhard Stock. Selecting features for markov modeling: A case study on hp35. *Journal of Chemical Theory and Computation*, 19(11):3391–3405, 2023.

[23] David K Wolfe, Joseph R Persichetti, Ajeet K Sharma, Phillip S Hudson, H Lee Woodcock, and Edward P O'Brien. Hierarchical markov state model building to describe molecular processes. *Journal of chemical theory and computation*, 16(3):1816–1826, 2020.

[24] Andreas Mardt, Luca Pasquali, Hao Wu, and Frank Noé. Vampnets for deep learning of molecular kinetics. *Nature communications*, 9(1):5, 2018.

[25] Diego E Kleiman and Diwakar Shukla. Active learning of the conformational ensemble of proteins using maximum entropy vampnets. *Journal of Chemical Theory and Computation*, 19(14):4377–4388, 2023.
